# Development of updated population norms for the SF-36 for Hungary and comparison with 1997–1998 norms

**DOI:** 10.1186/s12955-025-02343-5

**Published:** 2025-02-17

**Authors:** Alex Bató, Valentin Brodszky, Fanni Rencz

**Affiliations:** 1 Doctoral School, Semmelweis University, Budapest, Hungary; 2 Department of Health Policy, Corvinus University of Budapest, 8 Fővám Tér, Budapest, 1093 Hungary

**Keywords:** SF-36, Health-related quality of life, Population norm, Hungary

## Abstract

**Background:**

Hungarian SF-36 population norm data were last collected in 1997–1998 and have not been updated since, reducing their relevance and limiting their usability and comparability. This study aimed to establish contemporary normative data for the SF-36 domain and standardised summary scores in Hungary and compare them to the previous population norms.

**Methods:**

An online cross-sectional survey, including the SF-36v1, was conducted among 1,700 members of the Hungarian adult general population in 2020. The sample demonstrated good representativeness across key sociodemographic characteristics. Normative data were calculated for domains using raw scores and for summary scores using country-specific factor score coefficients derived from exploratory factor analysis. Multivariate linear regression models were performed to examine the association of domain and summary scores with sociodemographic and health-related characteristics. Raw domain scores were compared with the 1997–1998 norms.

**Results:**

Males reported higher scores (better health) in seven out of eight domains (*p* < 0.001). Mean standardised PCS scores decreased, whereas MCS scores increased with age (*p* < 0.001). Compared to the 1997–1998 population norms, the 18–24 and 25–34 age groups reported lower, while the 65 + age group reported higher scores in all eight domains. Higher scores were reported in 2020 from the 35–44 age group onward on the role physical, bodily pain, social functioning, and role emotional domains.

**Conclusions:**

This study established contemporary population norms for the SF-36 in Hungary. Our results highlight the changes in health status in the general population, particularly in young adults, compared to the 1997–1998 population norms, and provide valuable input to inform decision-makers.

**Supplementary Information:**

The online version contains supplementary material available at 10.1186/s12955-025-02343-5.

## Background

Health-related quality of life (HRQoL) refers to an individual’s perceived well-being in the physical, mental and social domains of health and functioning [1]. Measuring HRQoL of the general population is considered a key health metric, offering valuable information about the population’s overall health status to inform health and social policy decision-making. To measure an individual’s HRQoL, generic and condition-specific HRQoL measures are used [2]. Condition-specific measures focus on a particular target population and effectively capture a broad range of symptoms and health issues related to a specific condition (e.g. itching in skin-related conditions). Generic measures focus on health aspects relevant to various patient groups and the general population (e.g. physical, mental, and social dimensions of health). The 36‑item short form health survey (SF‑36) is one of the most commonly used generic HRQoL measures [3]. It is frequently included in observational studies with patient groups, population health studies, as an endpoint in clinical trials and in patient registries [4, 5]. SF-36 has been shown as a valid, reliable and responsive measure in multiple populations and is currently widely used both among the general population [6] and patients in most medical disciplines [7–10]. In Hungary, the SF-36 has been used in gastrointestinal, musculoskeletal and several patient groups, as well as to derive SF-6D scores [11–19].

Establishing normative values from a representative sample of the general population serves as a reference for interpreting the HRQoL of patient populations [20]. So far, SF-36 normative values have been developed for several countries [21–28]. In Hungary, population norms are available for a variety of generic HRQoL measures, including the EQ-5D-3L [29, 30] and EQ-5D-5L [31], SF-36v1 [32], PROPr [31], SF-6D [31], 15D [33], PROMIS-29 + 2 [34] and PROMIS-GH [35]. A recent study updated population norms for the EQ-5D-3L in Hungary after 22 years (2000 and 2022) and identified significant changes in the HRQoL of the population over time. Notably, there was an improvement in reporting problems related to pain/discomfort and anxiety/depression aged 35–64 years [29]. These differences may be related to the structural changes in factors related to the economy, society, culture, and the availability of health technologies over the past two decades. Hungarian SF-36 population norms have been available since 1999 (data collection: 1997–1998) [32]. However, these normative data, collected decades ago, have never been updated and may no longer be representative of the Hungarian general population, limiting their usability and comparability. In addition, the previous Hungarian SF-36 population norm study did not report country-specific weighting coefficients, further limiting its local relevance [36]. Therefore, our objective was to establish updated normative data for the eight SF-36 domains by age and gender and compare them to the 1997–1998 population norms. We further aimed to develop country-specific weighting coefficients and construct the two standardised summary scores of the SF-36 in Hungary. Moreover, we also aimed to explore the association of HRQoL as measured by the SF-36 with sociodemographic and health-related variables.

## Methods

### Study design and recruitment

The SF-36v1 was included in a longer survey aimed to assess the HRQoL and well-being of the general population in Hungary. In November 2020, an online cross-sectional survey was conducted involving 1,700 individuals from the Hungarian adult general population. The Research Ethics Committee of the Corvinus University of Budapest granted ethical approval for this study (no. KRH/343/2020). A survey company recruited participants from members of Hungary’s largest online panel. To approximate the demographic distribution of the general population, soft quotas were set for age, gender, education, place of residence, and geographical region. The criteria for inclusion in the study were: (i) ≥ 18 years of age; (ii) Hungarian residence; and (iii) giving informed consent prior to data collection. Participants were requested to provide information about their sociodemographic characteristics, including gender, age, education, place of residence, region, employment, household’s net monthly income, marital status, body weight, height, and any chronic health conditions. They also completed a set of standardised HRQoL and well-being measures, including EQ-5D-5L, PROMIS-29 + 2, PROMIS Global Health and the Satisfaction With Life Scale. Results related to other instruments have been reported elsewhere [13, 31, 34, 35, 37, 38].

### 36‑item short form health survey (SF‑36) measure

This study used the Hungarian version of the SF-36v1 with a four-week recall period [39]. SF-36 is a self-reported generic measure of HRQoL with 35 items that cover eight multi-item health domains, specifically physical functioning (PF, ten items), role limitations due to physical problems (RP, four items), bodily pain (BP, two items), general health (GH, five items), vitality (VT, four items), social functioning (SF, two items), role limitations due to emotional problems (RE, three items) and mental health (MH, five items). Furthermore, a single additional item assesses the change in perceived health over the past 12 months and is not included either in the domain scores or the summary scores [39, 40]. SF-36 allows the generation of two summary scores, one for physical health (PCS) that includes PF, RP, BP, and GH, and the other for mental health (MCS) including VT, SF, RE, and MH [41, 42].

### Statistical analyses

Normative data for the SF-36 were calculated using two approaches: (i) raw scores and (ii) country-specific scores. Raw domain and standardised summary scores were used to derive normative data presented by age, gender groups and other sociodemographic characteristics, and for the multivariate linear regressions. Raw domains scores were used to analyse floor and ceiling, to examine internal consistency and factor structure, and for comparisons with the 1997–1998 norms.

#### Normative data and psychometric properties of SF-36

First, the distribution of responses (frequency and relative frequency) for each of the 36 items was computed for the total sample and by age groups. Then, item responses were transformed to range from 0 to 100, where higher scores represent better HRQoL. Domain scores were computed by averaging the respective item scores. We calculated the mean and standard deviation of the raw domain and standardised summary scores. Normative data were calculated for the total sample and by age, gender groups and the following sociodemographic characteristics: education, place of residence, geographical region, employment, household’s net monthly income, marital status, body weight and height, and the presence of any chronic health conditions for the eight domains and two standardised summary scores. Shapiro–Wilk test was used to test the normality of the domain scores. Mann–Whitney and Kruskal–Wallis tests were used to compare differences across groups for domains, and independent samples t-test and ANOVA for standardised summary scores.

Floor and ceiling were assessed at the domain level by calculating the percentage of scores in each domain that reached the lowest and highest possible values. We considered floor or ceiling effects to be present if the relative frequency of respondents with the lowest and highest possible scores in a domain exceeded 15% [43]. The eight domains’ internal consistency reliability was examined using Cronbach’s alpha, and considered acceptable if > 0.80 [44].

#### Comparison of normative data between 2020 and 1997–1998

We compared the updated raw domain scores to the 1997–1998 normative data by gender and age groups [32]. Raw domain scores by place of residence were also reported earlier, but comparisons were not possible due to different groupings. Further comparison and testing were not possible as standard deviations were not reported in the 1997–1998 population norm study. Standardised summary scores could also not be compared as they were not reported in the previous population norm study.

#### Standardised summary scores of the SF-36

Country-specific standardised summary scores may be created for the SF-36 using factor analysis. Previous studies used either orthogonal or oblique rotation to derive country-specific scores for the SF-36 [41, 45–47]. Consequently, we derived both orthogonal and oblique factor score coefficients using exploratory factor analyses. Our research team decided to use the obliquely rotated two-factor model (allowing correlation between physical and mental health constructs) to generate the Hungarian population norms, as this approach tends to be less prone to produce inconsistencies [45, 46]. In addition, this methodological choice also allowed us to directly compare the Hungarian orthogonally rotated factor coefficients with the widely used US factor score coefficients [41]. Standardised summary scores were calculated by multiplying the z-score of each SF-36 domain by its respective scoring coefficient, followed by a transformation to ensure a mean of 50 and a standard deviation of 10 [41]. Values below and above 50 can be interpreted as a lower or higher HRQoL compared to the general population.

#### Predictors of raw SF-36 domain and standardised summary scores

Multivariate linear regression models were performed to explore the relationship of sociodemographic and health-related variables with the SF-36 raw domain and standardised summary scores. Coefficients and their 95% CIs were calculated for the same groups as described above for the normative data. For these analyses, the monthly net household income per capita was grouped according to the median income level in the sample (HUF 112,500). Statistical analysis was carried out in R Statistical Software (v4.3.0 Vienna, Austria). All statistical tests with a *p*-value < 0.05 were considered statistically significant [48].

## Results

### Sample characteristics

In total, 1,700 respondents completed the survey. The mean age was 47.9 ± 16.3 years, and 43.7% of the respondents were male. Approximately one-third of the sample had completed tertiary education (32.4%). Half of the respondents were employed (50.9%), while 23.5% were retired, and 4.4% were students. Overall, 24.1% of the sample reported no chronic disease. The overall sample showed good representativeness for the general population in Hungary; however, respondents with secondary education were slightly underrepresented, and those who lived in the capital were somewhat overrepresented (Table 1).

**Table 1:** Characteristics of the study population and SF-36 raw domain and standardised summary scores

Variables	General population (%) [49]	n (%)	PF		RP		BP		GH		VT		SF		RE		MH		Standardised PCS		Standardised MCS	
			**Mean**	**SD**	**Mean**	**SD**	**Mean**	**SD**	**Mean**	**SD**	**Mean**	**SD**	**Mean**	**SD**	**Mean**	**SD**	**Mean**	**SD**	**Mean**	**SD**	**Mean**	**SD**
**Total**	100	1,700 (100)	81.72	24.15	74.74	36.12	76.16	24.30	59.52	23.35	62.13	23.88	79.83	24.53	75.98	35.80	69.95	23.12	50.00	10.00	50.00	10.00
Female	53.1	957 (56.3)	79.35	25.68	71.29	37.57	72.73	25.60	59.23	23.78	58.55	24.53	77.06	25.64	71.93	37.83	66.94	24.12	49.63	10.79	49.16	10.64
Male	46.9	743 (43.7)	84.77	21.65	79.17	33.66	80.57	21.74	59.89	22.80	66.74	22.19	83.40	22.56	81.20	32.30	73.83	21.15	50.47	8.86	51.09	9.00
*p*-value*	-	-	< 0.001		< 0.001		< 0.001		0.714		< 0.001		< 0.001		< 0.001		< 0.001		0.085		< 0.001	
**Age groups (years)**																						
18–24	10.0	148 (8.7)	93.01	13.47	82.77	27.79	81.59	21.59	69.70	21.52	58.21	23.10	79.56	22.69	74.32	35.87	65.78	21.62	55.01	7.18	45.95	9.51
25–34	15.2	293 (17.2)	89.23	19.75	80.97	32.69	78.04	23.97	65.14	21.35	55.53	24.03	77.22	24.81	76.79	35.65	62.87	24.34	54.54	9.15	45.23	10.66
35–44	19.5	309 (18.2)	87.83	20.27	80.10	33.59	77.78	24.34	62.31	23.57	60.37	23.17	79.17	24.41	78.53	33.91	66.96	23.00	52.89	9.50	47.49	10.11
45–54	16.0	304 (17.9)	82.25	24.30	75.74	35.78	75.38	23.67	57.89	24.20	63.62	23.30	80.39	22.78	78.51	34.98	71.18	22.46	49.76	9.22	50.53	9.02
55–64	16.8	296 (17.4)	74.71	26.68	72.04	38.31	74.02	25.43	54.61	23.64	63.75	24.97	80.41	26.67	76.35	36.36	72.95	23.69	47.20	9.37	52.46	8.73
65 +	22.5	350 (20.6)	70.71	25.71	62.79	39.32	73.33	24.72	53.60	21.72	68.19	22.57	81.75	24.70	71.24	37.49	76.67	20.44	44.11	9.51	55.38	7.91
*p*-value*	-	-	< 0.001		< 0.001		0.001		< 0.001		< 0.001		0.033		0.035		< 0.001		< 0.001		< 0.001	
**Highest level of education**																						
Primary school or less	23.8	468 (27.5)	73.94	28.87	66.99	40.27	69.71	27.29	54.50	25.33	60.52	26.03	76.79	26.92	70.51	38.89	68.26	24.48	47.31	10.11	50.85	9.27
Secondary school	55.0	682 (40.1)	83.64	22.55	76.69	34.50	76.69	23.74	60.95	23.00	60.70	23.92	79.77	24.01	76.49	35.07	68.83	23.44	51.02	10.06	49.07	10.57
College/university degree	21.2	550 (32.4)	85.95	19.78	78.91	33.27	80.98	20.87	62.02	21.34	65.26	21.55	82.50	22.72	80.00	33.36	72.78	21.22	51.03	9.40	50.42	9.80
*p*-value*	-	-	< 0.001		< 0.001		< 0.001		< 0.001		0.002		0.005		< 0.001		0.009		< 0.001		0.006	
**Place of residence**																						
Capital	17.9	380 (22.4)	84.13	21.18	77.89	34.09	78.74	21.92	60.30	22.65	62.43	23.78	80.86	23.87	75.18	35.76	69.73	23.61	51.13	9.09	49.35	10.15
Other town	52.6	820 (48.2)	83.37	23.09	77.35	34.61	76.94	24.11	60.76	23.49	63.38	23.07	81.23	23.57	79.19	33.84	71.21	22.29	50.45	9.60	50.19	9.57
Village	29.5	500 (29.4)	77.17	27.20	68.05	39.10	72.90	25.98	56.88	23.49	59.84	25.11	76.75	26.28	71.33	38.40	68.06	23.96	48.41	11.07	50.18	10.56
*p*-value*	-	-	< 0.001		< 0.001		0.005		0.009		0.072		0.014		0.001		0.103		< 0.001		0.356	
**Geographical region**																						
Central Hungary	30.4	572 (33.6)	83.34	22.37	77.53	34.19	78.48	22.64	60.58	22.10	62.81	23.62	80.53	23.73	75.23	36.14	69.97	23.32	50.86	9.47	49.58	10.20
Eastern Hungary	39.5	635 (37.4)	80.28	25.42	72.99	37.24	74.33	25.12	58.31	24.12	60.75	24.20	77.95	25.65	74.80	36.14	69.04	23.48	49.52	10.60	49.91	10.36
Western Hungary	30.2	493 (29.0)	81.69	24.38	73.73	36.69	75.80	24.90	59.85	23.74	63.11	23.74	81.44	23.86	78.36	34.93	71.11	22.38	49.62	9.76	50.60	9.25
*p*-value*	-	-	0.400		0.107		0.042		0.261		0.149		0.078		0.136		0.404		0.039		0.246	
**Employment status**																						
Employed	53.1	865 (50.9)	87.92	18.65	82.08	31.06	79.54	21.95	62.97	22.20	61.83	23.15	82.11	22.50	80.69	32.49	70.25	22.43	52.44	8.43	48.79	9.68
Retired	26.1	399 (23.5)	70.28	26.77	63.28	39.42	73.21	24.73	53.11	21.75	67.71	22.75	81.02	25.17	71.09	37.88	76.00	21.05	44.25	9.78	55.09	8.25
Disability pensioner	3.1	67 (3.9)	49.85	32.50	39.18	44.85	53.99	29.45	33.81	23.63	46.04	28.39	61.19	32.79	49.75	43.18	55.82	28.18	40.47	11.82	50.17	11.37
Student	3.1	74 (4.4)	95.14	9.76	85.14	26.47	83.51	18.87	72.57	19.85	58.51	21.32	81.76	19.10	74.32	37.24	64.38	21.59	56.50	6.91	44.80	9.63
Unemployed	4.7	129 (7.6)	81.74	25.51	71.90	38.40	73.91	28.17	61.05	24.76	61.09	27.07	74.61	28.17	74.42	38.30	64.09	26.55	51.21	10.42	47.58	10.95
Homemaker/housewife	1.0	99 (5.8)	84.60	20.21	76.52	34.22	72.40	26.36	61.21	21.47	60.40	22.28	76.26	24.06	72.39	37.20	67.23	22.73	51.08	10.26	48.48	10.52
Other	0.0	67 (3.9)	82.54	23.79	75.00	35.62	73.88	23.93	58.96	24.29	57.31	24.11	75.19	25.33	80.60	32.39	65.61	22.30	51.21	9.12	47.86	9.11
*p*-value*	-	-	< 0.001		< 0.001		< 0.001		< 0.001		< 0.001		< 0.001		< 0.001		< 0.001		< 0.001		< 0.001	
Household net monthly income per person (HUF)**																						
0—66 779	n/a	224 (13.2)	75.96	28.90	66.29	40.97	70.11	28.54	53.55	26.97	54.80	26.79	69.92	28.61	66.22	39.87	59.21	26.72	50.04	10.95	46.47	10.64
66 780—99 511		252 (14.8)	75.34	28.93	65.38	40.65	69.46	25.66	55.69	24.98	57.16	25.18	75.50	26.19	66.27	40.74	67.17	23.57	47.53	11.12	50.25	10.14
99 512—126 924		229 (13.5)	80.11	25.36	74.24	36.60	75.39	25.32	60.28	23.09	63.78	23.09	81.11	24.15	78.46	33.94	72.44	22.89	48.98	9.97	51.36	9.64
126 925—164 049		207 (12.2)	84.23	20.88	76.93	33.63	77.19	23.09	60.75	22.62	61.86	24.23	80.86	24.69	76.97	34.78	70.90	23.59	50.56	9.47	49.96	10.36
164,050-		423 (24.9)	83.92	21.03	77.66	33.64	79.51	21.22	62.20	20.99	65.59	21.71	82.89	22.80	79.91	33.15	74.15	19.96	50.01	9.52	51.39	9.30
Don't know		69 (4.1)	89.78	15.84	82.61	29.49	82.03	20.52	62.54	20.80	63.77	21.19	79.35	24.24	75.85	35.19	69.74	22.25	52.96	7.58	48.39	9.58
Don't want to answer		296 (17.4)	85.96	20.40	81.93	31.33	80.14	22.55	61.30	22.45	65.47	22.56	85.05	19.37	83.45	30.69	71.91	21.42	51.76	9.29	49.82	9.84
*p*-value*	-	-	< 0.001		< 0.001		< 0.001		< 0.001		< 0.001		< 0.001		< 0.001		< 0.001		< 0.001		0.014	
**Marital status**																						
Married	45.6	718 (42.2)	81.05	24.29	75.00	35.50	76.72	24.12	59.09	23.66	64.03	23.51	81.69	24.37	76.42	35.40	73.08	22.24	49.04	9.55	51.51	9.19
Domestic partnership	13.4	360 (21.2)	85.26	21.38	77.01	34.42	73.90	25.26	60.46	21.89	59.39	23.16	78.47	23.57	79.35	33.14	67.32	22.49	51.54	9.95	48.26	10.32
Single	18.5	336 (19.8)	86.56	21.28	78.27	34.99	79.41	22.38	61.44	24.55	59.32	25.00	79.02	24.06	74.50	37.07	64.56	24.00	53.23	9.52	46.59	10.48
Widowed	11.4	98 (5.8)	67.65	26.67	65.05	41.36	73.83	24.73	56.58	22.48	67.86	23.31	78.57	28.38	70.75	40.69	75.22	21.43	44.42	10.88	54.83	9.10
Divorced	11.1	156 (9.2)	74.33	28.08	65.87	40.27	72.92	26.14	56.09	23.73	63.30	25.14	77.64	26.18	73.08	37.51	71.13	25.34	46.78	9.66	52.11	9.45
Other	n/a	32 (1.9)	85.00	25.21	78.91	31.82	77.66	22.75	63.91	17.95	56.56	15.58	76.56	21.71	73.96	34.64	64.00	19.12	53.07	9.32	46.41	8.73
*p*-value*	-	-	< 0.001		< 0.001		0.061		0.073		< 0.001		0.021		0.417		< 0.001		< 0.001		< 0.001	
**Chronic disease** [50]**																						
Yes	48.0	1146 (67.4)	76.31	25.60	67.02	38.74	70.95	24.85	53.05	22.49	58.70	24.20	76.90	25.89	70.16	38.15	67.95	23.55	47.79	10.41	50.40	10.18
No	52.0	410 (24.1)	94.40	14.62	92.38	20.94	88.48	18.12	75.84	18.20	71.56	21.60	87.26	19.62	89.15	25.56	76.58	20.84	54.61	6.68	50.00	9.19
Don’t know/Don’t want to answer	-	144 (8.5)	88.65	18.07	85.94	27.05	82.52	20.89	64.48	18.95	62.53	20.02	82.03	21.20	83.80	29.23	67.03	22.34	54.48	8.48	46.84	10.24
*p*-value*	-	-	< 0.001		< 0.001		< 0.001		< 0.001		< 0.001		< 0.001		< 0.001		< 0.001		< 0.001		< 0.001	
**BMI groups**																						
Underweight (under 18.5)	n/a	56 (3.3)	87.86	18.90	78.57	31.42	75.09	29.98	61.61	23.82	54.02	22.81	80.36	20.62	74.40	33.02	61.36	22.57	54.15	11.10	44.94	11.10
Normal weight (between 18.5 and 24.9)		497 (29.2)	86.90	20.40	77.92	34.51	78.96	22.86	63.59	24.10	62.15	23.15	79.63	23.76	77.46	35.32	69.22	23.12	51.92	9.45	48.75	10.07
Overweight (between 25 and 29.9)		535 (31.5)	83.95	22.21	76.03	35.74	77.74	23.52	60.49	22.90	64.20	24.35	82.99	23.48	77.51	34.78	72.82	22.59	49.95	9.20	50.96	9.58
Obesity (between 30 and 39.9)		374 (22.0)	73.36	26.02	68.25	38.13	72.19	24.90	54.02	22.17	62.11	23.79	79.28	25.05	72.55	37.83	71.28	22.78	46.73	10.41	52.14	9.73
*p*-value*	-	-	< 0.001		< 0.001		< 0.001		< 0.001		0.007		0.018		0.117		< 0.001		< 0.001		< 0.001	

*PF* physical functioning, *RP* role-physical, *BP* bodily pain, *GH* general health, *VT* vitality, *SF* social functioning, *RE* role-emotional, *MH* mental health, *MCS* mental component summary, *PCS* physical component summary, *SD* standard deviation, *BMI* body mass index (*n* = 238 were missing, *p*-value was computed without these respondents), *HUF* Hungarian forint, *n/a* = not available

^*^For domains, *p*-values were estimated using the Mann–Whitney test or the Kruskal–Wallis test. For standardised PCS and MCS, *p*-values were estimated using the independent samples t-test or ANOVA

^**^Responses ‘Don't know’ and ‘Don't want to answer’ were excluded when estimating the *p*-value

### Psychometric properties and normative data of SF-36

The distribution of responses to all SF-36 items for the total sample and by age group is provided in Additional Table 1. The proportion of respondents at the floor ranged between 0.4% (MH) and 12.7% (RP), indicating that no floor effects were found across the eight domains (Table 2). The ceiling ranged between 4.4% (GH) and 63.4% (RE). We found ceiling effects in the following five domains: RE (63.4%), RP (59.2%), SF (46.1%), PF (37.5%), and BP (33.8%). No floor and ceiling effects were observed for the GH, VT, and MH domains. We found acceptable internal consistency across the eight domains with Cronbach’s alpha values ranging from 0.885 (VT) to 0.893 (RE) (Table 2).

**Table 2:** Psychometric properties and country-specific standardised scoring coefficients of the SF-36

Domains	Hungary							US	
	**Floor % (n)**	**Ceiling % (n)**	**Cronbach’s alpha**	**Obliquely rotated factor score coefficients (PCS)**	**Obliquely rotated factor score coefficients (MCS)**	**Orthogonally rotated factor score coefficients (PCS)**	**Orthogonally rotated factor score coefficients (MCS)**	**Orthogonally rotated factor score coefficients (PCS)** [41]	**Orthogonally rotated factor score coefficients (MCS)** [41]
**Physical functioning (PF)**	0.82 (14)	37.47 (637)	0.891	0.4827	−0.3080	0.3376	−0.1116	0.42402	−0.22999
**Role-physical (RP)**	12.71 (216)	59.24 (1007)	0.886	0.5420	−0.2988	0.3961	−0.0815	0.35119	−0.12329
**Bodily pain (BP)**	0.53 (9)	33.76 (574)	0.886	0.2559	−0.1157	0.1963	−0.0149	0.31754	−0.09731
**General health (GH)**	0.76 (13)	4.35 (74)	0.890	0.1495	−0.0563	0.1188	0.0018	0.24954	−0.01571
**Vitality (VT)**	0.65 (11)	6.65 (113)	0.885	0.0448	0.0969	0.0771	0.1065	0.02877	0.23534
**Social functioning (SF)**	0.71 (12)	46.06 (783)	0.888	0.1250	−0.0375	0.1028	0.0105	−0.00753	0.26876
**Role-emotional (RE)**	12.18 (207)	63.35 (1077)	0.893	0.1046	−0.0420	0.0821	−0.0012	−0.19206	0.43407
**Mental health (MH)**	0.35 (6)	9.65 (164)	0.893	−0.9490	1.3751	−0.3832	0.9361	−0.22069	0.48581

*PCS* physical component summary, *MCS* mental component summary, *US* United States

Normative data by gender and age groups are presented in Table 1. In the total sample, mean domain scores ranged from 59.52 (GH) to 81.72 (PF). Except for the GH domain (*p* = 0.714), males reported significantly better HRQoL in all domains (*p* < 0.001). Mean raw scores for the four physical domains (PF, RP, BP, and GH) decreased, while for three of the four mental domains (VT, SF and MH) mean raw scores somewhat increased with advancing age (*p* < 0.05). Individuals living in villages reported, on average, lower scores than those living in the capital or other towns across all domains, except for VT and MH (*p* < 0.05).

### Normative data comparison between 2020 and 1997–1998

Comparisons of normative data are shown in Tables 3 and 4. The PF (1997–1998: 91, 2020: 81.72) and GH (1997–1998: 64, 2020: 59.52) domains accounted for the highest and lowest mean domain scores both in the 1997–1998 and 2020 population norms. When comparing the 1997–1998 and 2020 population norms, the largest absolute score difference was observed for females in the PF domain (1997–1998: 89, 2020: 79.35), and for males in the VT domain (1997–1998: 75, 2020: 66.74). From the age comparison point of view, the largest absolute difference between mean raw scores was observed in the VT domain 18–24 age group (1997–1998: 76, 2020: 58.21). Overall, the 18–24 and 25–34 age groups reported lower, while the 65 + age group reported higher mean scores across all eight domains in the 2020 population norms compared to 1997–1998. However, it is important to emphasise that the proportion of respondents aged over 65 years (2.9%) in the total sample of the 1997–1998 population study was substantially lower compared to our study (20.6%), particularly in males. Higher mean scores were reported in 2020 from the 35–44 age group onward on the RP, BP, SF, and RE domains. For three out of four PCS domains (RP, BP, GH), the largest absolute score differences were found in the 55–64 and 65 + age groups. While for the MCS domains, the largest absolute score differences for three out of four domains (VT, RE, MH) were found in the 18–24 age group. Additionally, while the mean raw scores of three MCS domains (VT, SF and MH) increased with age in the 2020 population norm, the opposite was observed in the 1997–1998 population norm study. Both in 1997–1998 and in 2020, females reported lower mean scores across all domains, except for GH in 2020, where females and males had almost identical mean scores.

**Table 3:** Comparison of population norms of the Hungarian SF-36

Variables	n (%)	n (%)	Mean PF raw score		Mean RP raw score		Mean BP raw score		Mean GH raw score		Mean VT raw score		Mean SF raw score		Mean RE raw score		Mean MH raw score	
	**2020**	**1997–1998**	**2020**	**1997–1998**	**2020**	**1997–1998**	**2020**	**1997–1998**	**2020**	**1997–1998**	**2020**	**1997–1998**	**2020**	**1997–1998**	**2020**	**1997–1998**	**2020**	**1997–1998**
**Total**	1,700 (100)	3907 (100)	81.72	91	74.74	79	76.16	78	59.52	64	62.13	70	79.83	80	75.98	78	69.95	71
Female	957 (56.3)	2381 (60.9)	79.35	89	71.29	76	72.73	76	59.23	62	58.55	67	77.06	78	71.93	74	66.94	69
Male	743 (43.7)	1526 (39.1)	84.77	93	79.17	83	80.57	81	59.89	67	66.74	75	83.40	84	81.20	85	73.83	76
**Age groups (years)**																		
18–24	148 (8.7)	646 (16.5)	93.01	96	82.77	90	81.59	84	69.70	73	58.21	76	79.56	85	74.32	84	65.78	77
25–34	293 (17.2)	983 (25.2)	89.23	95	80.97	84	78.04	81	65.14	67	55.53	73	77.22	82	76.79	82	62.87	72
35–44	309 (18.2)	1178 (30.2)	87.83	91	80.10	78	77.78	77	62.31	62	60.37	68	79.17	79	78.53	77	66.96	70
45–54	304 (17.9)	630 (16.1)	82.25	85	75.74	71	75.38	73	57.89	59	63.62	65	80.39	78	78.51	73	71.18	68
55–64	296 (17.4)	242 (6.2)	74.71	79	72.04	64	74.02	69	54.61	55	63.75	65	80.41	78	76.35	71	72.95	69
65 +	350 (20.6)	115 (2.9)	70.71	66	62.79	55	73.33	62	53.60	47	68.19	56	81.75	72	71.24	63	76.67	66

*PF* physical functioning, *RP* role-physical, *BP* bodily pain, *GH* general health, *VT* vitality, *SF* social functioning, *RE* role-emotional, *MH* mental health, *SD* standard deviation

1997–1998 population norms: [32]

**Table 4:** Comparison of SF-36 normative data by age and gender groups based on raw domain scores

Age groups (years)	Population norms	Gender groups	Statistics	PF	RP	BP	GH	VT	SF	RE	MH
**18–24**	**1997–1998**	**Female (*n* = 398)**	**Mean**	96	89	83	71	73	83	82	75
	**2020**	**Female (*n* = 115)**	**Mean**	92.13	81.74	79.35	67.43	55.78	78.37	73.62	63.79
			**SD**	14.20	27.77	22.60	21.71	22.80	23.72	35.73	21.26
	**1997–1998**	**Male (*n* = 248)**	**Mean**	97	91	86	76	81	89	88	81
	**2020**	**Male (*n* = 33)**	**Mean**	96.06	86.36	89.39	77.58	66.67	83.71	76.77	72.73
			**SD**	10.14	28.01	15.50	19.13	22.45	18.35	36.78	21.74
**25–34**	**1997–1998**	**Female (*n* = 624)**	**Mean**	94	81	79	66	70	80	78	70
	**2020**	**Female (*n* = 204)**	**Mean**	87.67	77.57	75.07	63.75	52.06	74.94	73.20	60.98
			**SD**	20.94	35.04	25.18	20.87	24.00	25.11	37.30	24.95
	**1997–1998**	**Male (*n* = 359)**	**Mean**	97	89	84	70	78	86	90	76
	**2020**	**Male (*n* = 89)**	**Mean**	92.81	88.76	84.83	68.31	63.48	82.44	85.02	67.19
			**SD**	16.27	25.00	19.39	22.22	22.24	23.44	30.16	22.43
**35–44**	**1997–1998**	**Female (*n* = 736)**	**Mean**	89	74	75	60	65	76	72	67
	**2020**	**Female (*n* = 168)**	**Mean**	85.45	75.89	74.32	61.58	58.45	76.56	76.19	64.62
			**SD**	22.05	35.56	26.89	24.45	23.62	25.85	34.83	24.22
	**1997–1998**	**Male (*n* = 442)**	**Mean**	93	83	81	66	74	83	86	75
	**2020**	**Male (*n* = 141)**	**Mean**	90.67	85.11	81.91	63.19	62.66	82.27	81.32	69.76
			**SD**	17.58	30.47	20.24	22.54	22.48	22.27	32.70	21.21
**45–54**	**1997–1998**	**Female (*n* = 379)**	**Mean**	82	67	69	57	62	75	69	64
	**2020**	**Female (*n* = 157)**	**Mean**	75.76	69.11	69.09	54.87	60.83	77.55	71.97	69.45
			**SD**	27.90	38.41	25.13	25.61	24.16	24.26	38.21	23.10
	**1997–1998**	**Male (*n* = 251)**	**Mean**	90	77	80	61	70	82	80	72
	**2020**	**Male (*n* = 147)**	**Mean**	89.18	82.82	82.09	61.12	66.60	83.42	85.49	73.03
			**SD**	17.31	31.35	20.00	22.23	22.03	20.75	29.73	21.67
**55–64**	**1997–1998**	**Female (*n* = 133)**	**Mean**	76	58	67	53	62	76	63	66
	**2020**	**Female (*n* = 158)**	**Mean**	71.99	67.09	71.11	55.19	61.20	77.85	70.25	70.84
			**SD**	27.00	40.31	25.54	25.40	26.75	28.04	39.92	25.93
	**1997–1998**	**Male (*n* = 109)**	**Mean**	84	72	72	57	67	80	80	73
	**2020**	**Male (*n* = 138)**	**Mean**	77.83	77.72	77.36	53.95	66.67	83.33	83.33	75.36
			**SD**	26.07	35.19	24.99	21.52	22.52	24.79	30.47	20.66
**65 + **	**1997–1998**	**Female (*n* = 59)**	**Mean**	59	50	58	43	50	68	56	62
	**2020**	**Female (*n* = 155)**	**Mean**	63.42	56.77	68.34	53.19	64.23	78.15	66.02	73.11
			**SD**	27.01	40.72	26.22	21.46	23.67	26.48	40.29	21.74
	**1997–1998**	**Male (*n* = 56)**	**Mean**	74	61	66	52	63	77	71	70
	**2020**	**Male (*n* = 195)**	**Mean**	76.51	67.56	77.29	53.92	71.33	84.62	75.38	79.51
			**SD**	23.10	37.60	22.76	21.98	21.20	22.86	34.65	18.93

*PF* physical functioning, *RP* role-physical, *BP* bodily pain, *GH* general health, *VT* vitality, *SF* social functioning, *RE* role-emotional, *MH* mental health, *SD* standard deviation

1997–1998 population norms: [32]

### Standardised country-specific PCS and MCS norm scores

The results of the exploratory factor analyses are shown in Table 2. PCS and MCS oblique factor score coefficients ranged from −0.9490 (MH) to 0.5420 (RP) and from −0.3080 (PF) to 1.3751 (MH), respectively. The absolute magnitude of the negative factor score coefficients was slightly greater for the oblique approach compared to those for the orthogonal approach for both PCS and MCS. In the oblique factor approach, all four physical domains had positive factor score coefficients for the physical factor. However, two mental domains had negative coefficients (SF and RE) for the mental factor. The Hungarian orthogonally rotated factor score coefficients were similar to those used in developing the US standardised summary scores. For PCS, the largest absolute difference was observed in the RE domain (Hungarian: 0.0821, US: −0.19206), while for MCS, it was observed in the MH domain (Hungarian: 0.9361, US: 0.48581) [41].

**Fig. 1:**
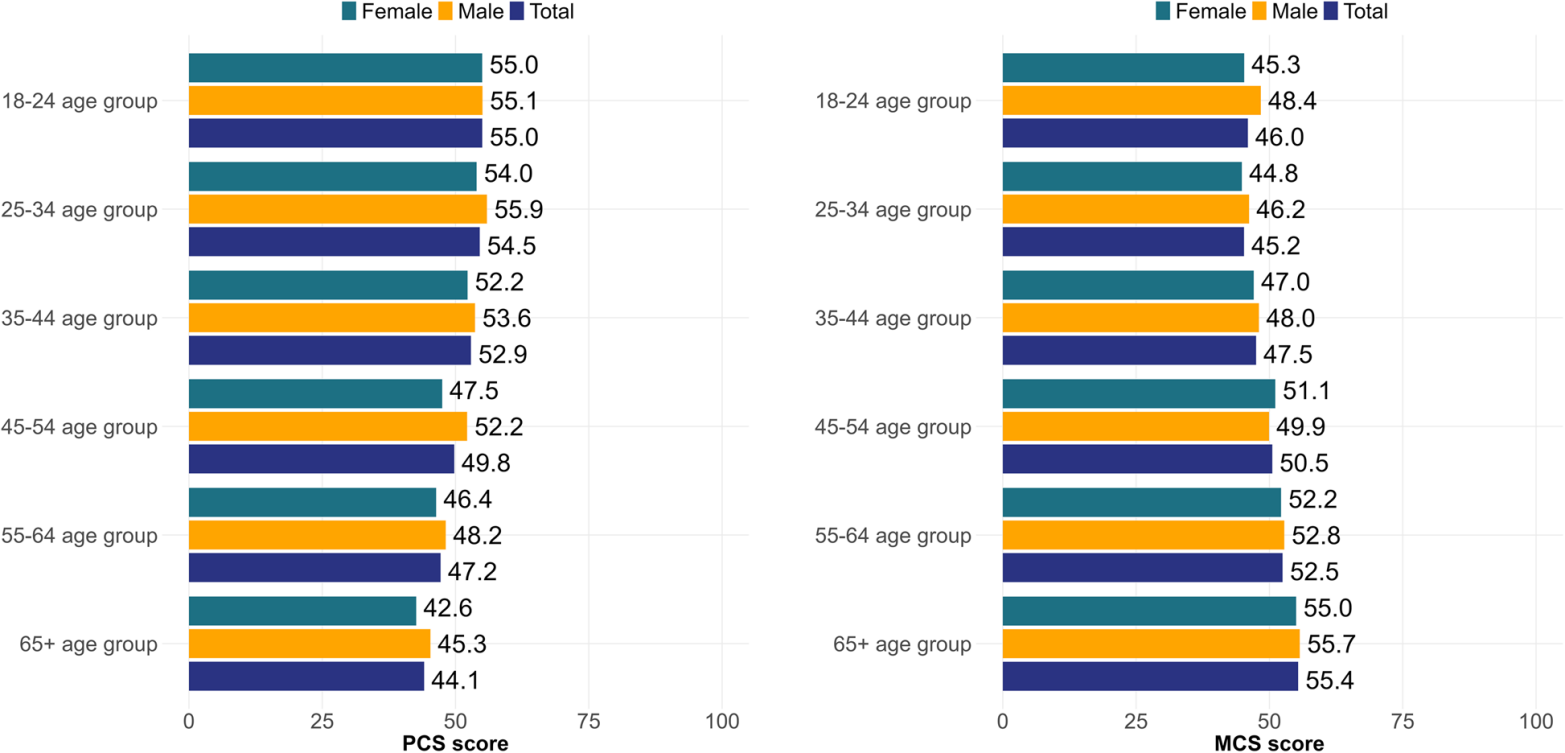
Country-specific standardised summary scores of the SF-36. PCS = physical component summary, MCS = mental component summary

The results showed that the mean PCS scores decreased with age, while the mean MCS scores increased with age (*p* < 0.001). Males had higher mean PCS and MCS scores in all age groups, except for MCS in the 45–54 age group, where the mean MCS score was 51.1 for females and 49.9 for males (Fig. 1). Overall, females had significantly lower MCS scores (49.16) compared to males (51.09) (*p* < 0.001). Those with higher levels of education or without any chronic disease reported higher PCS scores (*p* < 0.001). Mean PCS scores were higher in respondents living in the capital (*p* < 0.001) and in Central Hungary (*p* < 0.05). Students reported higher mean PCS scores, while retired respondents reported higher mean MCS scores (*p* < 0.001). In the case of marital status, single respondents reported the highest mean PCS scores, while widowed respondents reported the highest mean MCS scores (*p* < 0.001). Obese respondents had lower PCS and higher MCS scores than those with normal weight (*p* < 0.001) (Table 1).

### Multivariate regression model results

The results of regression models for the two standardised summary scores are shown in Table 5. Higher PCS scores were associated with male gender (*p* < 0.001). Older age was associated with lower PCS scores and higher MCS scores. The difference from the 18–24 age group was significant for both PCS and MCS from the 45 + age group (*p* < 0.05). Respondents with primary school education or less had lower PCS scores (*p* < 0.01). PCS scores were lower for retired respondents compared to those being employed (*p* < 0.01), and for disability pensioners (*p* < 0.001), while MCS scores were higher for retired respondents compared to those being employed (*p* < 0.01). Respondents with lower income had lower MCS scores compared to those with higher income (*p* < 0.05). Married and widowed respondents had higher MCS scores compared to those being single (*p* < 0.05). Obese respondents had lower PCS scores than those with normal weight (*p* < 0.01). The presence of any chronic diseases was associated with lower PCS and MCS scores (*p* < 0.001). Neither place of residence nor geographical region was associated with the summary scores. The regression results for the eight domain scores are presented in Additional Table 2.

**Table 5:** Multivariate linear regression of the two standardised summary scores of the SF-36

	**Standardised PCS**			**Standardised MCS**		
	**Coefficient**	**95% CI**	***p*-value**	**Coefficient**	**95% CI**	***p*-value**
**Intercept**	59.181	56.510, 61.852	< 0.001	45.983	42.936, 49.031	< 0.001
**Gender**						
Male^*a*^	-	-	-	-	-	-
Female	−1.761	−2.725, −0.798	< 0.001	−0.323	−1.362, 0.715	0.542
**Age groups (years)**						
18–24^*a*^	-	-	-	-	-	-
25–34	0.907	−1.227, 3.041	0.405	−1.974	−4.510, 0.563	0.127
35–44	−0.763	−2.990, 1.464	0.502	0.233	−2.366, 2.832	0.861
45–54	−2.750	−4.997, −0.502	0.017	3.098	0.517, 5.680	0.019
55–64	−4.041	−6.291, −1.791	< 0.001	4.578	2.002, 7.155	< 0.001
65 +	−5.186	−8.058, −2.315	< 0.001	5.556	2.462, 8.651	< 0.001
**Highest level of education**						
Primary school or less	−2.072	−3.324, −0.821	0.001	0.513	−0.810, 1.836	0.447
Secondary school	−0.508	−1.579, 0.563	0.352	−0.184	−1.343, 0.975	0.755
College/university degree^*a*^	-	-	-	-	-	-
**Place of residence**						
Capital^*a*^	-	-	-	-	-	-
Other town	0.999	−0.629, 2.626	0.229	0.312	−1.438, 2.062	0.727
Village	−0.555	−2.251, 1.141	0.521	0.183	−1.703, 2.069	0.849
**Geographical region**						
Central Hungary^*a*^	-	-	-	-	-	-
Eastern Hungary	−1.182	−2.720, 0.356	0.132	0.723	−0.888, 2.334	0.379
Western Hungary	−0.611	−2.169, 0.946	0.441	0.802	−0.807, 2.412	0.328
**Employment**						
Employed^*a*^	-	-	-	-	-	-
Retired	−3.040	−4.948, −1.131	0.002	2.411	0.630, 4.191	0.008
Disability pensioner	−6.522	−9.624, −3.419	< 0.001	−0.541	−3.505, 2.422	0.720
Student	0.562	−1.980, 3.104	0.665	−0.237	−3.373, 2.899	0.882
Unemployed	−1.403	−3.393, 0.588	0.167	−0.149	−2.334, 2.036	0.893
Homemaker/housewife	−1.709	−4.077, 0.659	0.157	2.232	−0.269, 4.732	0.080
Other	−1.141	−3.767, 1.484	0.393	−0.299	−3.039, 2.442	0.831
**Household net monthly income per person (HUF)**						
Lower median (≤ 125,001)^*a*^	-	-	-	-	-	-
Upper median (> 125,001)	−0.165	−1.295, 0.964	0.774	1.544	0.361, 2.727	0.011
Don’t know/Don’t want to answer	1.040	−0.175, 2.256	0.093	0.868	−0.475, 2.211	0.205
**Marital status**						
Married	−0.990	−2.340, 0.359	0.150	1.864	0.392, 3.336	0.013
Domestic partnership	−0.197	−1.646, 1.251	0.789	1.037	−0.611, 2.684	0.217
Single^*a*^	-	-	-	-	-	-
Widowed	−1.650	−4.148, 0.848	0.195	2.769	0.359, 5.180	0.024
Divorced	−0.805	−2.734, 1.125	0.414	1.309	−0.759, 3.377	0.214
Other	−0.936	−4.889, 3.017	0.642	2.383	−2.031, 6.798	0.290
**BMI groups**						
Underweight (under 18.5)	0.662	−2.051, 3.374	0.632	−2.001	−5.111, 1.110	0.207
Normal weight (between 18.5 and 24.9)^*a*^	-	-	-	-	-	-
Overweight (between 25 and 29.9)	−0.327	−1.423, 0.769	0.558	0.605	−0.581, 1.791	0.317
Obesity (between 30 and 39.9)	−2.043	−3.313, −0.774	0.002	1.100	−0.255, 2.455	0.112
**Chronic disease**						
Yes	−3.733	−4.695, −2.771	< 0.001	−2.363	−3.539, −1.187	< 0.001
No^*a*^	-	-	-	-	-	-
Don’t know/Don’t want to answer	0.319	−1.386, 2.024	0.714	−2.849	−4.821, −0.876	0.005

*PCS* physical component summary, *MCS* mental component summary, *CI* confidence intervals, *BMI* body mass index (*n* = 238 were missing, *p*-value was computed without these respondents), *HUF* Hungarian forint

^*a*^reference category

## Discussion

After more than two decades, this study provided updated population norms for the SF-36 in Hungary. The sample showed acceptable representativeness across all important socio-demographic groups. In terms of measurement properties, we found no floor effects for the eight domains, but found ceiling effects in several domains, as has been reported in previous general population studies in other countries [21, 25, 26]. Males reported significantly better HRQoL in all domains except for general health. The mean standardised scores for the PCS decreased with age, whereas MCS scores demonstrated an opposite trend.

Compared to the 1997–1998 population norms, worse HRQoL was reported across almost all age groups in the physical functioning and vitality domains. Generally better HRQoL was reported in 2020 from the 35–44 age group onward in the role physical, bodily pain, social functioning and role emotional domains. In particular, the 18–24 and 25–34 age groups reported worse HRQoL in the updated population norms compared to the 1997–1998 population norm study across all domains. Somewhat similar results were reported in a recent Hungarian EQ-5D-3L population norm study (year of data collection: 2022), where younger respondents reported worse mental health-related problems (e.g. anxiety/depression) than in the previous population norm data (year of data collection: 2000) [29]. Our sample was more representative of the older age group compared to the sample of the 1997–1998 population norm study. In addition, our study provided country-specific weighting coefficients for the two summary scores (PCS, MCS), as well as reported item-level distribution of responses by age groups, which have not been provided previously.

Regarding the normative data, one may assume that the HRQoL of the Hungarian general population has improved in the recent two decades, considering factors such as changes in healthy life expectancy at birth [51]. Over the past twenty years, profound structural transformations have occurred within the society, encompassing various factors such as the economy, culture, and health (e.g. the COVID-19 pandemic), which may be, at least, in part responsible for any changes in HRQoL of the population. However, there are some important differences between the two population norm studies. The 1997–1998 population norm data were collected in-person from patients and their relatives visiting general practitioners using a paper-and-pencil survey, while the 2020 survey was electronic and administered to members of an online panel. The population norms established in 1997–1998 were developed using responses only from individuals without any chronic health conditions; therefore, comparisons of normative data for chronic diseases were not possible. Another limitation regarding comparability is that the 1997–1998 population norm study was less representative of the Hungarian general population at that time compared to the 2020 study. For example, the 35–44 age group and older accounted for 55.4% of the sample size in the 1997–1998 study, compared to 74.1% in the 2020 population norm study.

One of the key findings of our study is the poorer HRQoL reported by young adults across most domains, coupled with an improvement in mental health as age increases. In terms of physical health, the observed trends may reflect increased health awareness and more open reporting of health issues. With respect to mental health, younger generations have experienced a significant rise in mental health challenges in recent years [52]. This may be attributed to the developmental stage of emerging adulthood, which is characterised by transitions in roles and responsibilities [53]. Furthermore, the widespread use of social media and constant digital connectivity has been linked to higher levels of anxiety, depression, sleep problems and reduced self-esteem among young adults [54–56]. Given that our sample was collected during the COVID-19 pandemic, it is also possible that stress resulting from disruptions to social life and daily activities contributed to the poorer mental health outcomes observed [57, 58].

This study has a few limitations. Our data were collected during the COVID-19 pandemic, which might have affected the HRQoL of the general population. Selection bias might have occurred as online panel data collection methods may lead to self-selection; for instance, those who participate in health surveys often report better health status than non-participants [59, 60]. Online panel data collection methods may also lead to the underrepresentation of certain groups, such as those without internet access. In addition, a non-probability quota sampling method was used, which may be considered another limitation for the generalisability of the results. Regarding the standardised summary scores, using country-specific factor score coefficients might limit comparisons with other studies that use different weights in other countries. However, we also reported raw scores, which still allow for inter-country comparisons. Future research is recommended to assess the clinimetric validity of the SF-36, building on previous examples [61–63].

In conclusion, this study provided updated age- and gender-specific domain and country-specific summary scores for the SF-36 in Hungary. Our results showed that the 18–24 and 25–34 age groups reported worse HRQoL in 2020 compared to the 1997–1998. These findings serve as valuable input for health and social policy decisions in Hungary and facilitate the comparability of the HRQoL of patients to the Hungarian general population. The SF-36 population norms established in the present study can serve as a useful reference point for any clinical application of the measure in Hungary, as they allow the comparison of patients’ detailed responses, domain, and summary scores with age- and gender-matched general population. Further research is needed to better understand the factors contributing to the deteriorated HRQoL in the young adult population.

## Supplementary Information

Additional file 1: Table 1 Distribution of SF-36 responses by item and age group. Table 2 Multivariate linear regression of the SF-36 raw domain scores.

## Data Availability

Data are available from the corresponding author upon a reasonable request.

## Abbreviations

BMI: Body mass index
BP: Bodily pain
CI: Confidence interval
GH: General health
HRQoL: Health-related quality of life
HUF: Hungarian forint
MCS: Mental component summary
MH: Mental health
n/a: Not available
PCS: Physical component summary
PF: Physical functioning
RE: Role-emotional
RP: Role-physical
SD: Standard deviation
SF: Social functioning
SF-36: 36‑Item short form health survey
US: United States
VT: Vitality
