# Supplementary material for: Development of updated population norms for the SF-36 for Hungary and comparison with 1997–1998 norms
Source: Health Qual Life Outcomes. 2025 Feb 17;23:14. doi: 10.1186/s12955-025-02343-5 (PMC11831779; doi:10.1186/s12955-025-02343-5)
Supplement: Supplementary file 1 — Additional file 1: Table 1 Distribution of SF-36 responses by item and age group. Table 2 Multivariate linear regression of the SF-36 raw domain scores. [file 12955_2025_2343_MOESM1_ESM.docx]

**Additional files**

**Development of updated population norms for the SF-36 for Hungary and comparison with 1997-1998 norms**

**Additional file 1**

**Additional table 1** Distribution of SF-36 responses by item and age group

**Additional table 2** Multivariate linear regression of the SF-36 raw domain scores

**Additional table 1 Distribution of SF-36 responses by item and age group**

| **Age groups (years)** | **18-24** | | **25-34** | | **35-44** | | **45-54** | | **55-64** | | **65+** | | **Total** | |
| --- | --- | --- | --- | --- | --- | --- | --- | --- | --- | --- | --- | --- | --- | --- |
|  | **n** | **%** | **n** | **%** | **n** | **%** | **n** | **%** | **n** | **%** | **n** | **%** | **n** | **%** |
| **In general, would you say your health is:** | | | | | | | | | | | | | | |
| Excellent | 29 | 19.6 | 37 | 12.6 | 32 | 10.4 | 17 | 5.6 | 11 | 3.7 | 13 | 3.7 | 139 | 8.2 |
| Very good | 44 | 29.7 | 90 | 30.7 | 88 | 28.5 | 79 | 26.0 | 45 | 15.2 | 55 | 15.7 | 401 | 23.6 |
| Good | 60 | 40.5 | 118 | 40.3 | 119 | 38.5 | 106 | 34.9 | 132 | 44.6 | 147 | 42.0 | 682 | 40.1 |
| Fair | 14 | 9.5 | 42 | 14.3 | 61 | 19.7 | 82 | 27.0 | 82 | 27.7 | 107 | 30.6 | 388 | 22.8 |
| Poor | 1 | 0.7 | 6 | 2.0 | 9 | 2.9 | 20 | 6.6 | 26 | 8.8 | 28 | 8.0 | 90 | 5.3 |
| **Compared to one year ago, how would you rate your health in general now?** | | | | | | | | | | | | | | |
| Much better now than one year ago | 22 | 14.9 | 30 | 10.2 | 27 | 8.7 | 15 | 4.9 | 14 | 4.7 | 11 | 3.1 | 119 | 7.0 |
| Somewhat better now than one year ago, | 22 | 14.9 | 38 | 13.0 | 30 | 9.7 | 27 | 8.9 | 19 | 6.4 | 23 | 6.6 | 159 | 9.4 |
| About the same as one year ago | 81 | 54.7 | 169 | 57.7 | 190 | 61.5 | 197 | 64.8 | 195 | 65.9 | 229 | 65.4 | 1061 | 62.4 |
| Somewhat worse now than one year ago | 23 | 15.5 | 50 | 17.1 | 54 | 17.5 | 52 | 17.1 | 58 | 19.6 | 77 | 22.0 | 314 | 18.5 |
| Much worse now than one year ago | 0 | 0.0 | 6 | 2.0 | 8 | 2.6 | 13 | 4.3 | 10 | 3.4 | 10 | 2.9 | 47 | 2.8 |
| **The following items are about activities you might do during a typical day. Does your health now limit you in these activities? Is so, how much?** | | | | | | | | | | | | | | |
| **Vigorous activities, such as running, lifting heavy objects, participating in strenuous sports** | | | | | | | | | | | | | | |
| Yes, limited a lot | 11 | 7.4 | 37 | 12.6 | 45 | 14.6 | 67 | 22.0 | 106 | 35.8 | 131 | 37.4 | 397 | 23.4 |
| Yes, limited a little | 39 | 26.4 | 72 | 24.6 | 81 | 26.2 | 97 | 31.9 | 111 | 37.5 | 160 | 45.7 | 560 | 32.9 |
| No, not limited at all | 98 | 66.2 | 184 | 62.8 | 183 | 59.2 | 140 | 46.1 | 79 | 26.7 | 59 | 16.9 | 743 | 43.7 |
| **Moderate activities, such as moving a table, pushing a vacuum cleaner, bowling, or playing golf** | | | | | | | | | | | | | | |
| Yes, limited a lot | 1 | 0.7 | 11 | 3.8 | 12 | 3.9 | 15 | 4.9 | 25 | 8.4 | 28 | 8.0 | 92 | 5.4 |
| Yes, limited a little | 12 | 8.1 | 39 | 13.3 | 41 | 13.3 | 72 | 23.7 | 78 | 26.4 | 126 | 36.0 | 368 | 21.6 |
| No, not limited at all | 135 | 91.2 | 243 | 82.9 | 256 | 82.8 | 217 | 71.4 | 193 | 65.2 | 196 | 56.0 | 1240 | 72.9 |
| **Lifting or carrying groceries** | | | | | | | | | | | | | | |
| Yes, limited a lot | 1 | 0.7 | 11 | 3.8 | 10 | 3.2 | 22 | 7.2 | 19 | 6.4 | 19 | 5.4 | 82 | 4.8 |
| Yes, limited a little | 10 | 6.8 | 35 | 11.9 | 38 | 12.3 | 51 | 16.8 | 71 | 24.0 | 111 | 31.7 | 316 | 18.6 |
| No, not limited at all | 137 | 92.6 | 247 | 84.3 | 261 | 84.5 | 231 | 76.0 | 206 | 69.6 | 220 | 62.9 | 1302 | 76.6 |
| **Climbing several flights of stairs** | | | | | | | | | | | | | | |
| Yes, limited a lot | 6 | 4.1 | 15 | 5.1 | 21 | 6.8 | 34 | 11.2 | 68 | 23.0 | 81 | 23.1 | 225 | 13.2 |
| Yes, limited a little | 25 | 16.9 | 56 | 19.1 | 69 | 22.3 | 85 | 28.0 | 103 | 34.8 | 152 | 43.4 | 490 | 28.8 |
| No, not limited at all | 117 | 79.1 | 222 | 75.8 | 219 | 70.9 | 185 | 60.9 | 125 | 42.2 | 117 | 33.4 | 985 | 57.9 |
| **Climbing one flight of stairs** | | | | | | | | | | | | | | |
| Yes, limited a lot | 1 | 0.7 | 7 | 2.4 | 10 | 3.2 | 14 | 4.6 | 23 | 7.8 | 32 | 9.1 | 87 | 5.1 |
| Yes, limited a little | 9 | 6.1 | 16 | 5.5 | 30 | 9.7 | 46 | 15.1 | 66 | 22.3 | 91 | 26.0 | 258 | 15.2 |
| No, not limited at all | 138 | 93.2 | 270 | 92.2 | 269 | 87.1 | 244 | 80.3 | 207 | 69.9 | 227 | 64.9 | 1355 | 79.7 |
| **Bending, kneeling, or stooping** | | | | | | | | | | | | | | |
| Yes, limited a lot | 2 | 1.4 | 16 | 5.5 | 16 | 5.2 | 24 | 7.9 | 44 | 14.9 | 52 | 14.9 | 154 | 9.1 |
| Yes, limited a little | 20 | 13.5 | 36 | 12.3 | 73 | 23.6 | 89 | 29.3 | 104 | 35.1 | 148 | 42.3 | 470 | 27.6 |
| No, not limited at all | 126 | 85.1 | 241 | 82.3 | 220 | 71.2 | 191 | 62.8 | 148 | 50.0 | 150 | 42.9 | 1076 | 63.3 |
| **Walking more than a mile** |  |  |  |  |  |  |  |  |  |  |  |  |  |  |
| Yes, limited a lot | 3 | 2.0 | 15 | 5.1 | 17 | 5.5 | 28 | 9.2 | 43 | 14.5 | 68 | 19.4 | 174 | 10.2 |
| Yes, limited a little | 12 | 8.1 | 39 | 13.3 | 36 | 11.7 | 55 | 18.1 | 67 | 22.6 | 101 | 28.9 | 310 | 18.2 |
| No, not limited at all | 133 | 89.9 | 239 | 81.6 | 256 | 82.8 | 221 | 72.7 | 186 | 62.8 | 181 | 51.7 | 1216 | 71.5 |
| **Walking several blocks** | | | | | | | | | | | | | | |
| Yes, limited a lot | 3 | 2.0 | 12 | 4.1 | 14 | 4.5 | 22 | 7.2 | 36 | 12.2 | 53 | 15.1 | 140 | 8.2 |
| Yes, limited a little | 9 | 6.1 | 24 | 8.2 | 29 | 9.4 | 40 | 13.2 | 48 | 16.2 | 65 | 18.6 | 215 | 12.6 |
| No, not limited at all | 136 | 91.9 | 257 | 87.7 | 266 | 86.1 | 242 | 79.6 | 212 | 71.6 | 232 | 66.3 | 1345 | 79.1 |
| **Walking one block** |  |  |  |  |  |  |  |  |  |  |  |  |  |  |
| Yes, limited a lot | 3 | 2.0 | 8 | 2.7 | 11 | 3.6 | 13 | 4.3 | 18 | 6.1 | 30 | 8.6 | 83 | 4.9 |
| Yes, limited a little | 5 | 3.4 | 23 | 7.8 | 19 | 6.1 | 33 | 10.9 | 41 | 13.9 | 55 | 15.7 | 176 | 10.4 |
| No, not limited at all | 140 | 94.6 | 262 | 89.4 | 279 | 90.3 | 258 | 84.9 | 237 | 80.1 | 265 | 75.7 | 1441 | 84.8 |
| **Bathing or dressing yourself** | | | | | | | | | | | | | | |
| Yes, limited a lot | 1 | 0.7 | 7 | 2.4 | 4 | 1.3 | 6 | 2.0 | 9 | 3.0 | 4 | 1.1 | 31 | 1.8 |
| Yes, limited a little | 2 | 1.4 | 13 | 4.4 | 16 | 5.2 | 21 | 6.9 | 26 | 8.8 | 45 | 12.9 | 123 | 7.2 |
| No, not limited at all | 145 | 98.0 | 273 | 93.2 | 289 | 93.5 | 277 | 91.1 | 261 | 88.2 | 301 | 86.0 | 1546 | 90.9 |
| **During the past 4 weeks, have you had any of the following problems with your work or other regular daily activities as a result of your physical health?** | | | | | | | | | | | | | | |
| **Cut down on the amount of time you spent on work or other activities** | | | | | | | | | | | | | | |
| Yes | 20 | 13.5 | 50 | 17.1 | 53 | 17.2 | 63 | 20.7 | 71 | 24.0 | 106 | 30.3 | 363 | 21.4 |
| No | 128 | 86.5 | 243 | 82.9 | 256 | 82.8 | 241 | 79.3 | 225 | 76.0 | 244 | 69.7 | 1337 | 78.6 |
| **Accomplished less than you would like** | | | | | | | | | | | | | | |
| Yes | 41 | 27.7 | 71 | 24.2 | 79 | 25.6 | 92 | 30.3 | 102 | 34.5 | 164 | 46.9 | 549 | 32.3 |
| No | 107 | 72.3 | 222 | 75.8 | 230 | 74.4 | 212 | 69.7 | 194 | 65.5 | 186 | 53.1 | 1151 | 67.7 |
| **Were limited in the kind of work or other activities** | | | | | | | | | | | | | | |
| Yes | 19 | 12.8 | 57 | 19.5 | 55 | 17.8 | 69 | 22.7 | 78 | 26.4 | 138 | 39.4 | 416 | 24.5 |
| No | 129 | 87.2 | 236 | 80.5 | 254 | 82.2 | 235 | 77.3 | 218 | 73.6 | 212 | 60.6 | 1284 | 75.5 |
| **Had difficulty performing the work or other activities (for example, it took extra effort)** | | | | | | | | | | | | | | |
| Yes | 22 | 14.9 | 45 | 15.4 | 59 | 19.1 | 71 | 23.4 | 80 | 27.0 | 113 | 32.3 | 390 | 22.9 |
| No | 126 | 85.1 | 248 | 84.6 | 250 | 80.9 | 233 | 76.6 | 216 | 73.0 | 237 | 67.7 | 1310 | 77.1 |
| **During the past 4 weeks, have you had any of the following problems with your work or other regular daily activities as a result of any emotional problems (such as feeling depressed or anxious)** | | | | | | | | | | | | | | |
| **Cut down on the amount of time you spent on work or other activities** | | | | | | | | | | | | | | |
| Yes | 29 | 19.6 | 61 | 20.8 | 57 | 18.4 | 53 | 17.4 | 62 | 20.9 | 88 | 25.1 | 350 | 20.6 |
| No | 119 | 80.4 | 232 | 79.2 | 252 | 81.6 | 251 | 82.6 | 234 | 79.1 | 262 | 74.9 | 1350 | 79.4 |
| **Accomplished less than you would like** | | | | | | | | | | | | | | |
| Yes | 41 | 27.7 | 70 | 23.9 | 79 | 25.6 | 79 | 26.0 | 91 | 30.7 | 125 | 35.7 | 485 | 28.5 |
| No | 107 | 72.3 | 223 | 76.1 | 230 | 74.4 | 225 | 74.0 | 205 | 69.3 | 225 | 64.3 | 1215 | 71.5 |
| **Didn’t do work or other activities as carefully as usual** | | | | | | | | | | | | | | |
| Yes | 44 | 29.7 | 73 | 24.9 | 63 | 20.4 | 64 | 21.1 | 57 | 19.3 | 89 | 25.4 | 390 | 22.9 |
| No | 104 | 70.3 | 220 | 75.1 | 246 | 79.6 | 240 | 78.9 | 239 | 80.7 | 261 | 74.6 | 1310 | 77.1 |
| **During the past 4 weeks, to what extent has your physical health or emotional problems interfered with your normal social activities with family, friends, neighbours, or groups?** | | | | | | | | | | | | | | |
| Not at all | 74 | 50.0 | 145 | 49.5 | 174 | 56.3 | 158 | 52.0 | 176 | 59.5 | 210 | 60.0 | 937 | 55.1 |
| Slightly | 38 | 25.7 | 65 | 22.2 | 70 | 22.7 | 79 | 26.0 | 62 | 20.9 | 81 | 23.1 | 395 | 23.2 |
| Moderately | 30 | 20.3 | 54 | 18.4 | 40 | 12.9 | 48 | 15.8 | 33 | 11.1 | 37 | 10.6 | 242 | 14.2 |
| Quite a bit | 4 | 2.7 | 21 | 7.2 | 16 | 5.2 | 17 | 5.6 | 18 | 6.1 | 15 | 4.3 | 91 | 5.4 |
| Extremely | 2 | 1.4 | 8 | 2.7 | 9 | 2.9 | 2 | 0.7 | 7 | 2.4 | 7 | 2.0 | 35 | 2.1 |
| **How much bodily pain have you had during the past 4 weeks?** | | | | | | | | | | | | | | |
| None | 69 | 46.6 | 117 | 39.9 | 116 | 37.5 | 97 | 31.9 | 89 | 30.1 | 103 | 29.4 | 591 | 34.8 |
| Very mild | 31 | 20.9 | 53 | 18.1 | 63 | 20.4 | 78 | 25.7 | 73 | 24.7 | 77 | 22.0 | 375 | 22.1 |
| Mild | 18 | 12.2 | 55 | 18.8 | 75 | 24.3 | 55 | 18.1 | 52 | 17.6 | 71 | 20.3 | 326 | 19.2 |
| Moderate | 23 | 15.5 | 48 | 16.4 | 34 | 11.0 | 54 | 17.8 | 52 | 17.6 | 74 | 21.1 | 285 | 16.8 |
| Severe | 6 | 4.1 | 16 | 5.5 | 16 | 5.2 | 19 | 6.3 | 29 | 9.8 | 20 | 5.7 | 106 | 6.2 |
| Very severe | 1 | 0.7 | 4 | 1.4 | 5 | 1.6 | 1 | 0.3 | 1 | 0.3 | 5 | 1.4 | 17 | 1.0 |
| **During the past 4 weeks, how much did pain interfere with your normal work (including both work outside the home and housework)?** | | | | | | | | | | | | | | |
| Not at all | 86 | 58.1 | 164 | 56.0 | 168 | 54.4 | 144 | 47.4 | 146 | 49.3 | 157 | 44.9 | 865 | 50.9 |
| A little bit | 43 | 29.1 | 80 | 27.3 | 88 | 28.5 | 91 | 29.9 | 84 | 28.4 | 112 | 32.0 | 498 | 29.3 |
| Moderately | 15 | 10.1 | 30 | 10.2 | 31 | 10.0 | 46 | 15.1 | 39 | 13.2 | 50 | 14.3 | 211 | 12.4 |
| Quite a bit | 3 | 2.0 | 14 | 4.8 | 12 | 3.9 | 21 | 6.9 | 18 | 6.1 | 26 | 7.4 | 94 | 5.5 |
| Extremely | 1 | 0.7 | 5 | 1.7 | 10 | 3.2 | 2 | 0.7 | 9 | 3.0 | 5 | 1.4 | 32 | 1.9 |
| **These questions are about how you feel and how things have been with you during the past 4 weeks. For each question, please give the one answer that comes closest to the way you have been feeling. How much of the time during the past 4 weeks —** | | | | | | | | | | | | | | |
| **Did you feel full of pep?** | | | | | | | | | | | | | | |
| All of the time | 22 | 14.9 | 36 | 12.3 | 46 | 14.9 | 53 | 17.4 | 43 | 14.5 | 68 | 19.4 | 268 | 15.8 |
| Most of the time | 42 | 28.4 | 85 | 29.0 | 92 | 29.8 | 97 | 31.9 | 105 | 35.5 | 144 | 41.1 | 565 | 33.2 |
| A good bit of the time | 31 | 20.9 | 52 | 17.7 | 64 | 20.7 | 58 | 19.1 | 66 | 22.3 | 58 | 16.6 | 329 | 19.4 |
| Some of the time | 31 | 20.9 | 59 | 20.1 | 61 | 19.7 | 56 | 18.4 | 41 | 13.9 | 47 | 13.4 | 295 | 17.4 |
| A little of the time | 13 | 8.8 | 40 | 13.7 | 18 | 5.8 | 29 | 9.5 | 20 | 6.8 | 20 | 5.7 | 140 | 8.2 |
| None of the time | 9 | 6.1 | 21 | 7.2 | 28 | 9.1 | 11 | 3.6 | 21 | 7.1 | 13 | 3.7 | 103 | 6.1 |
| **Have you been a very nervous person?** | | | | | | | | | | | | | | |
| All of the time | 0 | 0.0 | 7 | 2.4 | 9 | 2.9 | 7 | 2.3 | 9 | 3.0 | 3 | 0.9 | 35 | 2.1 |
| Most of the time | 14 | 9.5 | 32 | 10.9 | 19 | 6.1 | 8 | 2.6 | 11 | 3.7 | 12 | 3.4 | 96 | 5.6 |
| A good bit of the time | 19 | 12.8 | 62 | 21.2 | 48 | 15.5 | 39 | 12.8 | 26 | 8.8 | 21 | 6.0 | 215 | 12.6 |
| Some of the time | 46 | 31.1 | 77 | 26.3 | 66 | 21.4 | 63 | 20.7 | 44 | 14.9 | 51 | 14.6 | 347 | 20.4 |
| A little of the time | 36 | 24.3 | 61 | 20.8 | 88 | 28.5 | 97 | 31.9 | 86 | 29.1 | 73 | 20.9 | 441 | 25.9 |
| None of the time | 33 | 22.3 | 54 | 18.4 | 79 | 25.6 | 90 | 29.6 | 120 | 40.5 | 190 | 54.3 | 566 | 33.3 |
| **Have you felt so down in the dumps that nothing could cheer you up?** | | | | | | | | | | | | | | |
| All of the time | 2 | 1.4 | 4 | 1.4 | 5 | 1.6 | 3 | 1.0 | 5 | 1.7 | 1 | 0.3 | 20 | 1.2 |
| Most of the time | 8 | 5.4 | 23 | 7.8 | 9 | 2.9 | 7 | 2.3 | 10 | 3.4 | 8 | 2.3 | 65 | 3.8 |
| A good bit of the time | 15 | 10.1 | 23 | 7.8 | 35 | 11.3 | 17 | 5.6 | 19 | 6.4 | 14 | 4.0 | 123 | 7.2 |
| Some of the time | 21 | 14.2 | 35 | 11.9 | 40 | 12.9 | 40 | 13.2 | 22 | 7.4 | 34 | 9.7 | 192 | 11.3 |
| A little of the time | 30 | 20.3 | 44 | 15.0 | 40 | 12.9 | 47 | 15.5 | 40 | 13.5 | 42 | 12.0 | 243 | 14.3 |
| None of the time | 72 | 48.6 | 164 | 56.0 | 180 | 58.3 | 190 | 62.5 | 200 | 67.6 | 251 | 71.7 | 1057 | 62.2 |
| **Have you felt calm and peaceful?** | | | | | | | | | | | | | | |
| All of the time | 24 | 16.2 | 33 | 11.3 | 51 | 16.5 | 68 | 22.4 | 71 | 24.0 | 94 | 26.9 | 341 | 20.1 |
| Most of the time | 25 | 16.9 | 80 | 27.3 | 89 | 28.8 | 87 | 28.6 | 103 | 34.8 | 127 | 36.3 | 511 | 30.1 |
| A good bit of the time | 41 | 27.7 | 54 | 18.4 | 59 | 19.1 | 55 | 18.1 | 44 | 14.9 | 45 | 12.9 | 298 | 17.5 |
| Some of the time | 29 | 19.6 | 49 | 16.7 | 56 | 18.1 | 45 | 14.8 | 35 | 11.8 | 39 | 11.1 | 253 | 14.9 |
| A little of the time | 14 | 9.5 | 44 | 15.0 | 29 | 9.4 | 30 | 9.9 | 20 | 6.8 | 18 | 5.1 | 155 | 9.1 |
| None of the time | 15 | 10.1 | 33 | 11.3 | 25 | 8.1 | 19 | 6.3 | 23 | 7.8 | 27 | 7.7 | 142 | 8.4 |
| **Did you have a lot of energy?** | | | | | | | | | | | | | | |
| All of the time | 18 | 12.2 | 23 | 7.8 | 43 | 13.9 | 50 | 16.4 | 37 | 12.5 | 49 | 14.0 | 220 | 12.9 |
| Most of the time | 29 | 19.6 | 70 | 23.9 | 67 | 21.7 | 74 | 24.3 | 91 | 30.7 | 106 | 30.3 | 437 | 25.7 |
| A good bit of the time | 38 | 25.7 | 71 | 24.2 | 79 | 25.6 | 76 | 25.0 | 62 | 20.9 | 76 | 21.7 | 402 | 23.6 |
| Some of the time | 35 | 23.6 | 59 | 20.1 | 58 | 18.8 | 60 | 19.7 | 45 | 15.2 | 56 | 16.0 | 313 | 18.4 |
| A little of the time | 10 | 6.8 | 39 | 13.3 | 28 | 9.1 | 21 | 6.9 | 26 | 8.8 | 29 | 8.3 | 153 | 9.0 |
| None of the time | 18 | 12.2 | 31 | 10.6 | 34 | 11.0 | 23 | 7.6 | 35 | 11.8 | 34 | 9.7 | 175 | 10.3 |
| **Have you felt downhearted and blue?** | | | | | | | | | | | | | | |
| All of the time | 4 | 2.7 | 17 | 5.8 | 10 | 3.2 | 5 | 1.6 | 9 | 3.0 | 7 | 2.0 | 52 | 3.1 |
| Most of the time | 12 | 8.1 | 41 | 14.0 | 36 | 11.7 | 14 | 4.6 | 16 | 5.4 | 12 | 3.4 | 131 | 7.7 |
| A good bit of the time | 23 | 15.5 | 36 | 12.3 | 37 | 12.0 | 41 | 13.5 | 31 | 10.5 | 27 | 7.7 | 195 | 11.5 |
| Some of the time | 38 | 25.7 | 57 | 19.5 | 60 | 19.4 | 60 | 19.7 | 52 | 17.6 | 51 | 14.6 | 318 | 18.7 |
| A little of the time | 34 | 23.0 | 68 | 23.2 | 63 | 20.4 | 76 | 25.0 | 63 | 21.3 | 82 | 23.4 | 386 | 22.7 |
| None of the time | 37 | 25.0 | 74 | 25.3 | 103 | 33.3 | 108 | 35.5 | 125 | 42.2 | 171 | 48.9 | 618 | 36.4 |
| **Did you feel worn out?** | | | | | | | | | | | | | | |
| All of the time | 8 | 5.4 | 13 | 4.4 | 8 | 2.6 | 6 | 2.0 | 11 | 3.7 | 6 | 1.7 | 52 | 3.1 |
| Most of the time | 11 | 7.4 | 36 | 12.3 | 31 | 10.0 | 22 | 7.2 | 24 | 8.1 | 22 | 6.3 | 146 | 8.6 |
| A good bit of the time | 28 | 18.9 | 64 | 21.8 | 51 | 16.5 | 52 | 17.1 | 28 | 9.5 | 29 | 8.3 | 252 | 14.8 |
| Some of the time | 45 | 30.4 | 63 | 21.5 | 71 | 23.0 | 63 | 20.7 | 57 | 19.3 | 65 | 18.6 | 364 | 21.4 |
| A little of the time | 26 | 17.6 | 65 | 22.2 | 79 | 25.6 | 74 | 24.3 | 86 | 29.1 | 88 | 25.1 | 418 | 24.6 |
| None of the time | 30 | 20.3 | 52 | 17.7 | 69 | 22.3 | 87 | 28.6 | 90 | 30.4 | 140 | 40.0 | 468 | 27.5 |
| **Have you been a happy person?** | | | | | | | | | | | | | | |
| All of the time | 31 | 20.9 | 45 | 15.4 | 51 | 16.5 | 61 | 20.1 | 47 | 15.9 | 80 | 22.9 | 315 | 18.5 |
| Most of the time | 32 | 21.6 | 72 | 24.6 | 70 | 22.7 | 81 | 26.6 | 89 | 30.1 | 98 | 28.0 | 442 | 26.0 |
| A good bit of the time | 31 | 20.9 | 54 | 18.4 | 56 | 18.1 | 41 | 13.5 | 58 | 19.6 | 59 | 16.9 | 299 | 17.6 |
| Some of the time | 27 | 18.2 | 54 | 18.4 | 69 | 22.3 | 60 | 19.7 | 50 | 16.9 | 43 | 12.3 | 303 | 17.8 |
| A little of the time | 16 | 10.8 | 36 | 12.3 | 27 | 8.7 | 33 | 10.9 | 20 | 6.8 | 33 | 9.4 | 165 | 9.7 |
| None of the time | 11 | 7.4 | 32 | 10.9 | 36 | 11.7 | 28 | 9.2 | 32 | 10.8 | 37 | 10.6 | 176 | 10.4 |
| **Did you feel tired?** | | | | | | | | | | | | | | |
| All of the time | 9 | 6.1 | 19 | 6.5 | 16 | 5.2 | 13 | 4.3 | 14 | 4.7 | 11 | 3.1 | 82 | 4.8 |
| Most of the time | 18 | 12.2 | 45 | 15.4 | 33 | 10.7 | 26 | 8.6 | 26 | 8.8 | 23 | 6.6 | 171 | 10.1 |
| A good bit of the time | 32 | 21.6 | 68 | 23.2 | 62 | 20.1 | 62 | 20.4 | 46 | 15.5 | 43 | 12.3 | 313 | 18.4 |
| Some of the time | 40 | 27.0 | 78 | 26.6 | 76 | 24.6 | 77 | 25.3 | 65 | 22.0 | 80 | 22.9 | 416 | 24.5 |
| A little of the time | 26 | 17.6 | 48 | 16.4 | 65 | 21.0 | 72 | 23.7 | 95 | 32.1 | 110 | 31.4 | 416 | 24.5 |
| None of the time | 23 | 15.5 | 35 | 11.9 | 57 | 18.4 | 54 | 17.8 | 50 | 16.9 | 83 | 23.7 | 302 | 17.8 |
| **During the past 4 weeks, how much of the time has your physical health or emotional problems interfered with your social activities (like visiting with friends, relatives, etc.)?** | | | | | | | | | | | | | | |
| All of the time | 4 | 2.7 | 11 | 3.8 | 12 | 3.9 | 10 | 3.3 | 17 | 5.7 | 18 | 5.1 | 72 | 4.2 |
| Most of the time | 12 | 8.1 | 20 | 6.8 | 24 | 7.8 | 15 | 4.9 | 22 | 7.4 | 22 | 6.3 | 115 | 6.8 |
| Some of the time | 22 | 14.9 | 56 | 19.1 | 64 | 20.7 | 57 | 18.8 | 47 | 15.9 | 49 | 14.0 | 295 | 17.4 |
| A little of the time | 28 | 18.9 | 50 | 17.1 | 33 | 10.7 | 44 | 14.5 | 26 | 8.8 | 47 | 13.4 | 228 | 13.4 |
| None of the time | 82 | 55.4 | 156 | 53.2 | 176 | 57.0 | 178 | 58.6 | 184 | 62.2 | 214 | 61.1 | 990 | 58.2 |
| **How TRUE or FALSE is each of the following statements for you?** | | | | | | | | | | | | | | |
| **I seem to get sick a little easier than other people** | | | | | | | | | | | | | | |
| Definitely true | 4 | 2.7 | 10 | 3.4 | 12 | 3.9 | 10 | 3.3 | 7 | 2.4 | 6 | 1.7 | 49 | 2.9 |
| Mostly true | 11 | 7.4 | 29 | 9.9 | 30 | 9.7 | 27 | 8.9 | 21 | 7.1 | 20 | 5.7 | 138 | 8.1 |
| Don’t know | 24 | 16.2 | 49 | 16.7 | 48 | 15.5 | 60 | 19.7 | 56 | 18.9 | 76 | 21.7 | 313 | 18.4 |
| Mostly false | 41 | 27.7 | 78 | 26.6 | 82 | 26.5 | 80 | 26.3 | 91 | 30.7 | 119 | 34.0 | 491 | 28.9 |
| Definitely false | 68 | 45.9 | 127 | 43.3 | 137 | 44.3 | 127 | 41.8 | 121 | 40.9 | 129 | 36.9 | 709 | 41.7 |
| **I am as healthy as anybody I know** | | | | | | | | | | | | | | |
| Definitely true | 51 | 34.5 | 63 | 21.5 | 70 | 22.7 | 66 | 21.7 | 56 | 18.9 | 54 | 15.4 | 360 | 21.2 |
| Mostly true | 38 | 25.7 | 111 | 37.9 | 108 | 35.0 | 95 | 31.3 | 85 | 28.7 | 108 | 30.9 | 545 | 32.1 |
| Don’t know | 36 | 24.3 | 76 | 25.9 | 67 | 21.7 | 66 | 21.7 | 78 | 26.4 | 88 | 25.1 | 411 | 24.2 |
| Mostly false | 16 | 10.8 | 27 | 9.2 | 43 | 13.9 | 48 | 15.8 | 46 | 15.5 | 60 | 17.1 | 240 | 14.1 |
| Definitely false | 7 | 4.7 | 16 | 5.5 | 21 | 6.8 | 29 | 9.5 | 31 | 10.5 | 40 | 11.4 | 144 | 8.5 |
| **I expect my health to get worse** | | | | | | | | | | | | | | |
| Definitely true | 2 | 1.4 | 8 | 2.7 | 13 | 4.2 | 16 | 5.3 | 19 | 6.4 | 18 | 5.1 | 76 | 4.5 |
| Mostly true | 10 | 6.8 | 48 | 16.4 | 62 | 20.1 | 66 | 21.7 | 77 | 26.0 | 97 | 27.7 | 360 | 21.2 |
| Don’t know | 39 | 26.4 | 67 | 22.9 | 66 | 21.4 | 87 | 28.6 | 61 | 20.6 | 75 | 21.4 | 395 | 23.2 |
| Mostly false | 26 | 17.6 | 74 | 25.3 | 81 | 26.2 | 67 | 22.0 | 90 | 30.4 | 116 | 33.1 | 454 | 26.7 |
| Definitely false | 71 | 48.0 | 96 | 32.8 | 87 | 28.2 | 68 | 22.4 | 49 | 16.6 | 44 | 12.6 | 415 | 24.4 |
| **My health is excellent** | | | | | | | | | | | | | | |
| Definitely true | 40 | 27.0 | 46 | 15.7 | 53 | 17.2 | 34 | 11.2 | 21 | 7.1 | 19 | 5.4 | 213 | 12.5 |
| Mostly true | 47 | 31.8 | 121 | 41.3 | 101 | 32.7 | 94 | 30.9 | 82 | 27.7 | 93 | 26.6 | 538 | 31.6 |
| Don’t know | 24 | 16.2 | 57 | 19.5 | 45 | 14.6 | 52 | 17.1 | 37 | 12.5 | 52 | 14.9 | 267 | 15.7 |
| Mostly false | 22 | 14.9 | 39 | 13.3 | 69 | 22.3 | 62 | 20.4 | 68 | 23.0 | 83 | 23.7 | 343 | 20.2 |
| Definitely false | 15 | 10.1 | 30 | 10.2 | 41 | 13.3 | 62 | 20.4 | 88 | 29.7 | 103 | 29.4 | 339 | 19.9 |

**Additional table 2 Multivariate linear regression of the SF-36 raw domain scores**

| **Variables** | **Physical functioning (PF)** | | | **Social functioning (SF)** | | | **Role-physical (RP)** | | | **Bodily pain (BP)** | | | **Mental health (MH)** | | | **Role-emotional (RE)** | | | **Vitality (VT)** | | | **General health (GH)** | | |
| --- | --- | --- | --- | --- | --- | --- | --- | --- | --- | --- | --- | --- | --- | --- | --- | --- | --- | --- | --- | --- | --- | --- | --- | --- |
|  | **ß** | **95% CI** | **p-value** | **ß** | **95% CI** | **p-value** | **ß** | **95% CI** | **p-value** | **ß** | **95% CI** | **p-value** | **ß** | **95% CI** | **p-value** | **ß** | **95% CI** | **p-value** | **ß** | **95% CI** | **p-value** | **ß** | **95% CI** | **p-value** |
| **Intercept** | 104.775 | 98.972, 110.579 | <0.001 | 86.974 | 79.272, 94.676 | <0.001 | 97.971 | 87.851, 108.090 | <0.001 | 99.756 | 92.515, 106.995 | <0.001 | 71.967 | 64.860, 79.075 | <0.001 | 79.236 | 67.684, 90.788 | <0.001 | 66.845 | 59.113, 74.577 | <0.001 | 77.797 | 70.549, 85.045 | <0.001 |
| **Gender** | | | | | | | | | | | | | | | | | | | | | | | | |
| Male | - | - | - | - | - | - | - | - | - | - | - | - | - | - | - | - | - | - | - | - | - | - | - | - |
| Female | -6.334 | -8.615, -4.053 | <0.001 | -3.315 | -5.945, -0.685 | 0.014 | -7.638 | -11.343, -3.933 | <0.001 | -6.749 | -9.185, -4.313 | <0.001 | -3.528 | -5.962, -1.094 | 0.004 | -8.012 | -11.825, -4.199 | <0.001 | -5.684 | -8.185, -3.184 | <0.001 | -0.259 | -2.582, 2.063 | 0.827 |
| **Age groups (years)** | | | | | | | | | | | | | | | | | | | | | | | | |
| 18-24 | - | - | - | - | - | - | - | - | - | - | - | - | - | - | - | - | - | - | - | - | - | - | - | - |
| 25-34 | -1.743 | -6.351, 2.864 | 0.458 | -2.749 | -9.484, 3.986 | 0.423 | -0.739 | -8.936, 7.458 | 0.860 | -2.834 | -8.939, 3.270 | 0.363 | -4.929 | -10.843, 0.986 | 0.102 | 4.741 | -4.800, 14.281 | 0.330 | -1.455 | -7.876, 4.966 | 0.657 | -1.620 | -7.571, 4.332 | 0.594 |
| 35-44 | -3.446 | -8.195, 1.304 | 0.155 | -0.546 | -7.363, 6.271 | 0.875 | -1.530 | -9.925, 6.865 | 0.721 | -4.658 | -10.960, 1.644 | 0.147 | -1.070 | -7.086, 4.946 | 0.727 | 6.652 | -3.090, 16.393 | 0.181 | 3.403 | -3.065, 9.870 | 0.302 | -3.341 | -9.458, 2.775 | 0.284 |
| 45-54 | -4.980 | -9.881, -0.078 | 0.046 | 1.539 | -5.351, 8.430 | 0.661 | -2.033 | -10.859, 6.792 | 0.651 | -4.464 | -10.861, 1.932 | 0.171 | 4.372 | -1.707, 10.451 | 0.159 | 9.725 | -0.214, 19.664 | 0.055 | 9.068 | 2.409, 15.726 | 0.008 | -6.283 | -12.529, -0.036 | 0.049 |
| 55-64 | -9.999 | -15.190, -4.808 | <0.001 | 2.978 | -4.199, 10.156 | 0.416 | -1.200 | -10.197, 7.798 | 0.794 | -4.306 | -10.840, 2.228 | 0.196 | 6.561 | 0.382, 12.741 | 0.037 | 10.270 | 0.057, 20.482 | 0.049 | 11.043 | 4.225, 17.861 | 0.002 | -5.963 | -12.278, 0.355 | 0.064 |
| 65+ | -10.580 | -17.791, -3.370 | 0.004 | 4.112 | -4.524, 12.748 | 0.350 | -6.861 | -18.112, 4.389 | 0.232 | -5.506 | -13.563, 2.551 | 0.180 | 7.607 | 0.117, 15.096 | 0.047 | 6.539 | -6.002, 19.079 | 0.307 | 12.459 | 4.329, 20.589 | 0.003 | -4.170 | -11.688, 3.349 | 0.277 |
| **Highest level of education** | | | | | | | | | | | | | | | | | | | | | | | | |
| Primary school or less | -6.184 | -9.118, -3.250 | <0.001 | -0.786 | -4.223, 2.651 | 0.654 | -5.987 | -10.999, -0.976 | 0.019 | -6.841 | -10.130, -3.553 | <0.001 | -1.675 | -4.831, 1.481 | 0.298 | -4.767 | -9.832, 0.298 | 0.065 | -2.329 | -5.582, 0.925 | 0.160 | -3.108 | -6.219, 0.003 | 0.050 |
| Secondary school | -1.784 | -4.064, 0.496 | 0.125 | -0.647 | -3.434, 2.140 | 0.649 | -1.413 | -5.310, 2.484 | 0.477 | -3.341 | -5.953, -0.729 | 0.012 | -1.208 | -3.794, 1.379 | 0.360 | -1.684 | -5.824, 2.456 | 0.425 | -2.113 | -4.754, 0.529 | 0.117 | -1.382 | -3.894, 1.130 | 0.281 |
| College/ university degree | - | - | - | - | - | - | - | - | - | - | - | - | - | - | - | - | - | - | - | - | - | - | - | - |
| **Place of residence** | | | | | | | | | | | | | | | | | | | | | | | | |
| Capital | - | - | - | - | - | - | - | - | - | - | - | - | - | - | - | - | - | - | - | - | - | - | - | - |
| Other town | 2.672 | -1.094, 6.438 | 0.164 | 0.147 | -4.247, 4.542 | 0.948 | 5.452 | -0.797, 11.702 | 0.087 | 3.404 | -0.808, 7.615 | 0.113 | 2.383 | -1.708, 6.475 | 0.253 | 5.566 | -1.440, 12.571 | 0.119 | 2.860 | -1.485, 7.206 | 0.197 | 4.554 | 0.771, 8.338 | 0.018 |
| Village | -1.811 | -5.945, 2.322 | 0.390 | -4.434 | -9.178, 0.310 | 0.067 | -2.274 | -8.850, 4.302 | 0.498 | 0.414 | -3.990, 4.818 | 0.854 | -0.321 | -4.742, 4.100 | 0.887 | -1.618 | -8.896, 5.658 | 0.663 | -0.382 | -4.996, 4.232 | 0.871 | 1.794 | -2.139, 5.727 | 0.371 |
| **Geographical region** | | | | | | | | | | | | | | | | | | | | | | | | |
| Central Hungary | - | - | - | - | - | - | - | - | - | - | - | - | - | - | - | - | - | - | - | - | - | - | - | - |
| Eastern Hungary | -0.806 | -4.404, 2.793 | 0.661 | 0.156 | -3.936, 4.248 | 0.940 | -3.508 | -9.279, 2.264 | 0.233 | -3.743 | -7.662, 0.176 | 0.061 | 0.581 | -3.112, 4.273 | 0.758 | 0.171 | -6.161, 6.504 | 0.958 | -0.892 | -4.741, 2.956 | 0.649 | -2.386 | -5.751, 0.979 | 0.164 |
| Western Hungary | 0.874 | -2.716, 4.464 | 0.633 | 3.539 | -0.485, 7.563 | 0.085 | -1.869 | -7.712, 3.973 | 0.530 | -2.327 | -6.274, 1.620 | 0.248 | 1.525 | -2.162, 5.212 | 0.417 | 3.342 | -3.047, 9.732 | 0.305 | 0.899 | -2.972, 4.771 | 0.649 | -1.284 | -4.709, 2.142 | 0.462 |
| **Employment** |  | | | | | | | | | | | | | | | | | | | | | | | |
| Employed | - | - | - | - | - | - | - | - | - | - | - | - | - | - | - | - | - | - | - | - | - | - | - | - |
| Retired | -6.537 | -11.827, -1.246 | 0.015 | -0.977 | -6.224, 4.270 | 0.715 | -7.329 | -14.608, -0.050 | 0.048 | -0.897 | -5.904, 4.111 | 0.725 | 2.238 | -2.169, 6.644 | 0.319 | -2.190 | -9.650, 5.270 | 0.565 | 3.294 | -1.414, 8.002 | 0.170 | -5.097 | -9.449, -0.746 | 0.022 |
| Disability pensioner | -21.983 | -29.953, -14.013 | <0.001 | -14.359 | -23.053, -5.665 | 0.001 | -28.573 | -40.849, -16.298 | <0.001 | -14.773 | -22.470, -7.075 | <0.001 | -11.719 | -19.022, -4.416 | 0.002 | -18.676 | -30.075, -7.277 | 0.001 | -12.205 | -19.725, -4.686 | 0.001 | -19.000 | -25.595, -12.406 | <0.001 |
| Student | 1.817 | -3.034, 6.668 | 0.463 | 0.647 | -6.714, 8.007 | 0.863 | -1.244 | -11.467, 8.980 | 0.811 | -1.575 | -8.534, 5.384 | 0.657 | -0.328 | -7.492, 6.835 | 0.928 | 1.203 | -10.713, 13.118 | 0.843 | 3.175 | -4.374, 10.724 | 0.409 | 6.670 | -0.556, 13.955 | 0.070 |
| Unemployed | -4.926 | -9.335, -0.517 | 0.029 | -6.305 | -11.811, -0.800 | 0.025 | -8.213 | -15.706, -0.719 | 0.032 | -4.253 | -9.470, 0.964 | 0.110 | -3.232 | -8.364, 1.900 | 0.217 | -2.392 | -10.050, 5.265 | 0.540 | 1.816 | -3.237, 6.869 | 0.481 | -0.897 | -5.346, 3.552 | 0.693 |
| Homemaker/housewife | -1.075 | -5.812, 3.662 | 0.656 | -1.177 | -6.604, 4.249 | 0.670 | -0.822 | -8.460, 6.813 | 0.833 | -2.084 | -7.945, 3.777 | 0.486 | 3.746 | -1.564, 9.056 | 0.167 | -4.099 | -12.605, 4.407 | 0.345 | 7.019 | 1.954, 12.083 | 0.007 | 0.131 | -4.865, 5.127 | 0.959 |
| Other | -5.600 | -11.468, 0.269 | 0.061 | -4.796 | -12.100, 2.508 | 0.198 | -4.562 | -14.600, 5.475 | 0.373 | -2.514 | -8.373, 3.344 | 0.400 | -2.704 | -9.024, 3.616 | 0.401 | 5.080 | -4.192, 14.352 | 0.283 | -2.983 | -9.342, 3.376 | 0.358 | -4.935 | -10.866, 0.995 | 0.103 |
| **Household net monthly income per person (HUF)** | | | | | | | | | | | | | | | | | | | | | | | | |
| Lower median (≤ 125,001) | - | - | - | - | - | - | - | - | - | - | - | - | - | - | - | - | - | - | - | - | - | - | - | - |
| Upper median (> 125,001) | 1.549 | -0.934, 4.031 | 0.221 | 2.876 | -0.053, 5.804 | 0.054 | 3.222 | -1.039, 7.483 | 0.138 | 2.522 | -0.309, 5.353 | 0.081 | 4.380 | 1.669, 7.091 | 0.002 | 4.033 | -0.290, 8.356 | 0.067 | 3.433 | 0.684, 6.183 | 0.014 | 3.191 | 0.560, 5.821 | 0.017 |
| Don’t know/Don’t want to answer | 3.347 | 0.601, 6.094 | 0.017 | 5.860 | 2.802, 8.918 | <0.001 | 7.296 | 2.707, 11.886 | 0.002 | 4.387 | 1.196, 7.578 | 0.007 | 4.045 | 0.961, 7.129 | 0.010 | 7.083 | 2.371, 11.794 | 0.003 | 4.465 | 1.371, 7.558 | 0.005 | 2.271 | -0.690, 5.232 | 0.133 |
| **Marital status** | | | | | | | | | | | | | | | | | | | | | | | | |
| Married | 0.123 | -2.826, 3.071 | 0.935 | 1.237 | -2.413, 4.887 | 0.506 | 1.382 | -3.621, 6.386 | 0.588 | -0.274 | -3.633, 3.084 | 0.873 | 4.405 | 1.044, 7.765 | 0.010 | 4.135 | -1.177, 9.447 | 0.127 | 0.825 | -2.664, 4.313 | 0.643 | 2.639 | -0.603, 5.882 | 0.111 |
| Domestic partnership | 1.450 | -1.511, 4.411 | 0.337 | 1.508 | -2.261, 5.276 | 0.433 | 3.242 | -2.108, 8.592 | 0.235 | -2.467 | -6.121, 1.188 | 0.186 | 3.019 | -0.674, 6.712 | 0.109 | 9.518 | 3.957, 15.079 | <0.001 | 1.233 | -2.544, 5.010 | 0.522 | 1.540 | -1.886, 4.967 | 0.378 |
| Single | - | - | - | - | - | - | - | - | - | - | - | - | - | - | - | - | - | - | - | - | - | - | - | - |
| Widowed | -3.100 | -8.877, 2.676 | 0.293 | -0.902 | -7.345, 5.541 | 0.784 | 1.502 | -8.050, 11.053 | 0.758 | 1.614 | -4.259, 7.486 | 0.590 | 5.661 | 0.235, 11.086 | 0.041 | 3.841 | -5.920, 13.602 | 0.440 | 4.244 | -1.499, 9.987 | 0.147 | 5.328 | 0.223, 10.426 | 0.041 |
| Divorced | 0.274 | -4.535, 5.083 | 0.911 | -1.571 | -6.923, 3.781 | 0.565 | -0.608 | -8.322, 7.106 | 0.877 | -0.209 | -5.235, 4.818 | 0.935 | 2.929 | -2.104, 7.963 | 0.254 | 5.058 | -2.547, 12.662 | 0.192 | 0.592 | -4.517, 5.702 | 0.820 | 2.766 | -1.932, 7.463 | 0.248 |
| Other | -2.014 | -10.621, 6.593 | 0.646 | -0.823 | -9.221, 7.576 | 0.848 | 6.128 | -5.865, 18.120 | 0.316 | 2.579 | -7.624, 12.783 | 0.620 | 6.010 | -3.132, 15.152 | 0.197 | 7.237 | -8.967, 23.442 | 0.381 | 1.543 | -6.197, 9.282 | 0.696 | 4.859 | -2.907, 12.626 | 0.220 |
| **BMI groups** | | | | | | | | | | | | | | | | | | | | | | | | |
| Underweight (under 18.5) | -1.065 | -5.701, 3.570 | 0.652 | 1.752 | -4.145, 7.650 | 0.560 | -1.229 | -9.344, 6.887 | 0.767 | -3.962 | -11.338, 3.414 | 0.292 | -4.926 | -11.581, 1.729 | 0.147 | -0.493 | -9.691, 8.705 | 0.916 | -4.799 | -11.159, 1.561 | 0.139 | -5.719 | -11.685, 0.247 | 0.060 |
| Normal weight (between 18.5 and 24.9) | - | - | - | - | - | - | - | - | - | - | - | - | - | - | - | - | - | - | - | - | - | - | - | - |
| Overweight (between 25 and 29.9) | -0.228 | -2.633, 2.177 | 0.853 | 2.989 | 0.128, 5.849 | 0.041 | 0.594 | -3.562, 4.750 | 0.780 | -0.122 | -2.818, 2.573 | 0.929 | 1.435 | -1.294, 4.165 | 0.302 | -0.317 | -4.514, 3.879 | 0.882 | -0.296 | -3.080, 2.489 | 0.835 | 0.198 | -2.474, 2.869 | 0.885 |
| Obesity (between 30 and 39.9) | -7.052 | -9.974, -4.131 | <0.001 | 0.601 | -2.736, 3.940 | 0.724 | -2.924 | -7.680, 1.831 | 0.228 | -3.472 | -6.574, -0.370 | 0.028 | 0.147 | -3.002, 3.295 | 0.927 | -2.395 | -7.248, 2.458 | 0.333 | -1.750 | -4.925, 1.424 | 0.280 | -3.221 | -6.111, -0.331 | 0.029 |
| **Chronic disease** | | | | | | | | | | | | | | | | | | | | | | | | |
| Yes | -11.230 | -13.309, -9.152 | <0.001 | -11.092 | -13.738, -8.445 | <0.001 | -20.376 | -23.749, -17.002 | <0.001 | -15.406 | -17.900, -12.912 | <0.001 | -12.025 | -14.719, -9.330 | <0.001 | -17.694 | -21.434, -13.955 | <0.001 | -15.233 | -17.990, -12.477 | <0.001 | -20.440 | -22.917, -17.963 | <0.001 |
| No | - | - | - | - | - | - | - | - | - | - | - | - | - | - | - | - | - | - | - | - | - | - | - | - |
| Don’t know/Don’t want to answer | -3.286 | -6.488, -0.083 | 0.044 | -1.917 | -5.888, 2.055 | 0.344 | -5.239 | -10.939, 0.461 | 0.072 | -3.681 | -7.976, 0.614 | 0.093 | -7.960 | -12.242, -3.678 | <0.001 | -4.736 | -11.068, 1.596 | 0.143 | -7.472 | -11.593, -3.352 | <0.001 | -10.350 | -14.608, -6.092 | <0.001 |

ß = coefficient, CI = confidence intervals, BMI = body mass index (n=238 were missing, p-value was computed without these respondents), HUF = Hungarian forint
